# Supplementary material for: Transcriptomic diversity in human medullary thymic epithelial cells
Source: Nat Commun. 2022 Aug 2;13:4296. doi: 10.1038/s41467-022-31750-1 (PMC9345899; doi:10.1038/s41467-022-31750-1)
Supplement: Supplementary file 1 — Supplementary Information [file 41467_2022_31750_MOESM1_ESM.pdf]

Transcriptomic diversity in human medullary thymic epithelial cells

Jason A. Carter[1,2,3], Léonie Strömich[4,5], Matthew Peacey[6], Sarah R. Chapin[1], Lars Velten[7,8], Lars M. Steinmetz[9,10], Benedikt Brors[4], Sheena Pinto[4], and Hannah V. Meyer[1,*]

[1]Simons Center for Quantitative Biology, Cold Spring Harbor Laboratory, Cold Spring Harbor, NY, USA
[2]Medical Scientist Training Program, Stony Brook University, Stony Brook, NY, USA
[3]Department of Surgery, University of Washington, Seattle, WA, USA
[4]German Cancer Research Center, Heidelberg, Germany
[5]current affiliation: Imperial College London, London, UK
[6]School of Biological Sciences, Cold Spring Harbor Laboratory, Cold Spring Harbor, NY, USA
[7]Centre for Genomic Regulation, The Barcelona Institute of Science and Technology, Barcelona, Spain
[8]Universitat Pompeu Fabra (UPF), Barcelona, Spain
[9]European Molecular Biology Laboratory, Genome Biology Unit, Heidelberg, Germany
[10]Department of Genetics, Stanford University School of Medicine, Stanford, CA, USA Stanford Genome Technology Center, Palo Alto, CA, USA
[*]To whom correspondence should be addressed. E-mail: hmeyer@cshl.edu

1 Supplementary Figures

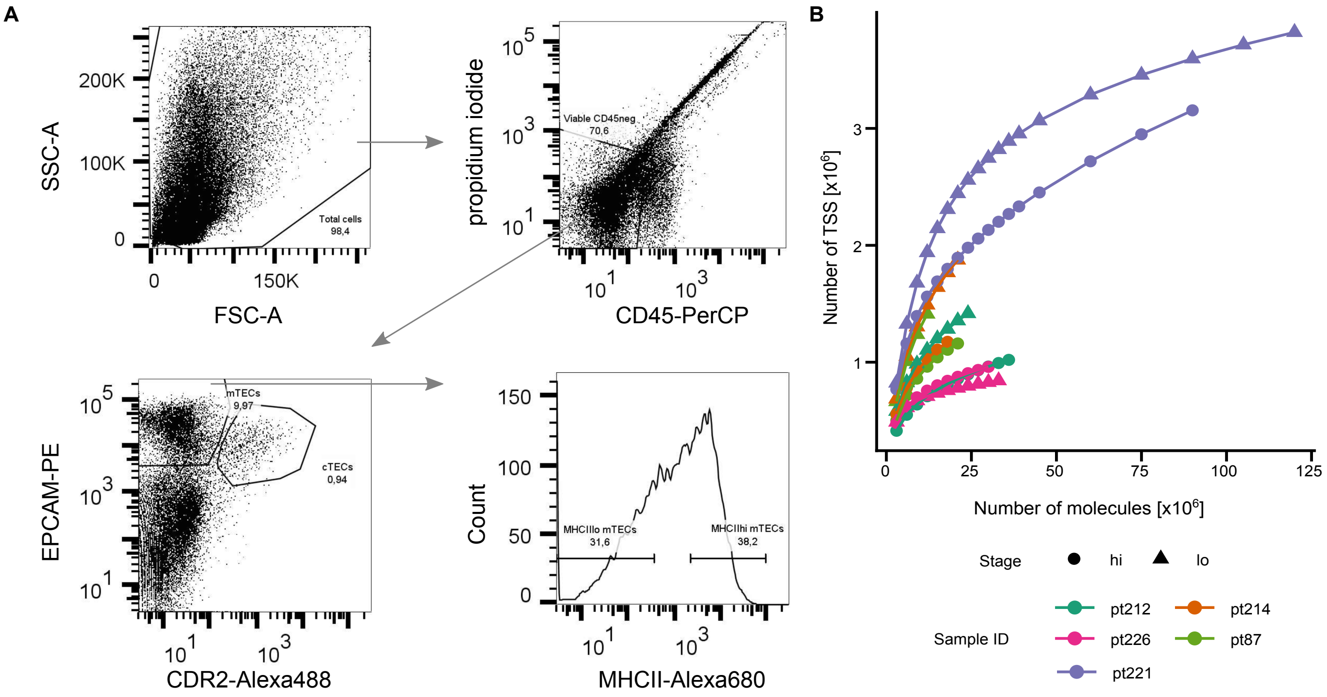

Supplementary Figure 1. Generation of transcription start site (TSS) data from human mTEC. (A) Sorting strategy to obtain mTEC^{hi} and mTEC^{lo} populations: Forward (FSC-A) and side scatter (SSC-A) to select all cells (upper left); gate on viable, CD45- cells (everything but hematopoietic; upper right); gate on EPCAM high, CDR2 (cortical TEC marker) negative cells to select for mTECs (lower left); gate for mTEC^{lo} and mTEC^{hi} populations based on their MHCII expression (lower right). **(B.)** Number of recovered TSS in relation to the number of sequenced molecules per library. We down-sampled each patient sample (mTEC^{lo} and mTEC^{hi}) to the same number of molecules per library and applied our TSS calling approach across a sequence of molecule thresholds, with the maximum at the total library size per sample. Upward-pointing trajectories indicate that more TSS could still be detected at deeper sequencing; n=5 paired mTEC samples. Source data for panel B is provided in the Source Data file.

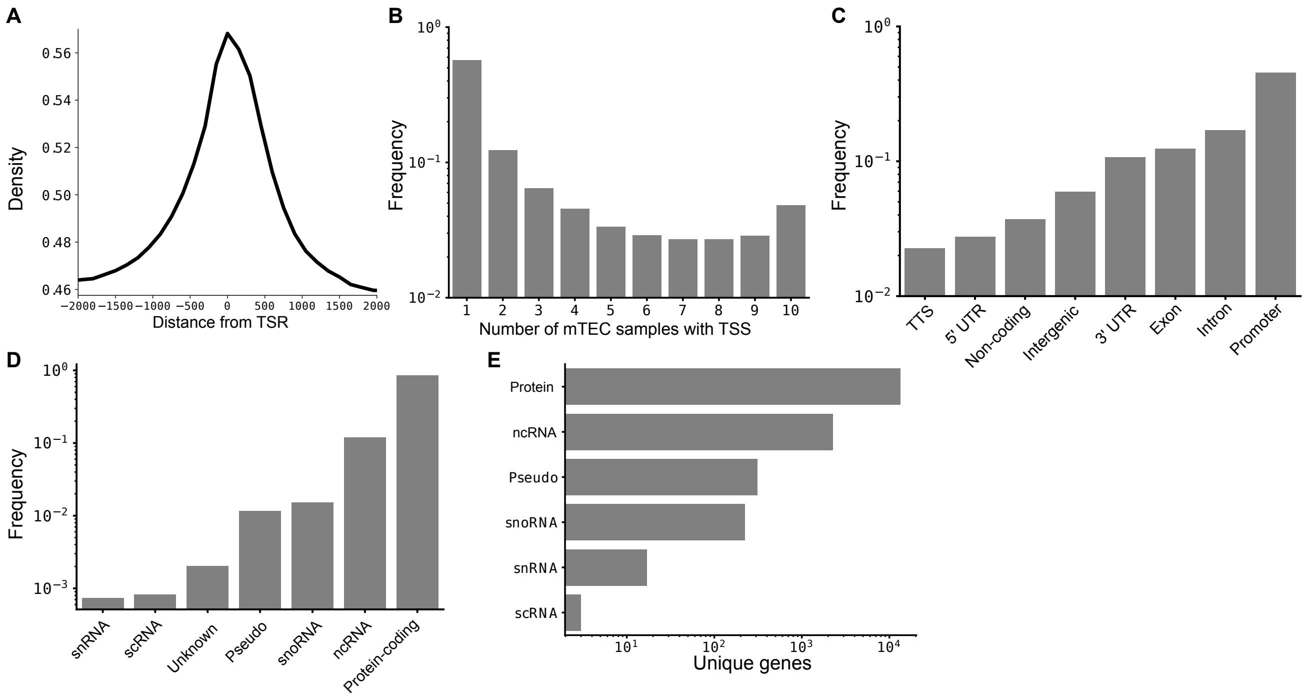

Supplementary Figure 2. Characteristics of transcription start regions (TSRs) in 10 mTEC samples. **(A)** GC nucleotide content around union of TSRs identified in all mTECs. **(B)** Frequency at which TSRs are shared between mTEC samples. **(C)** Frequencies of genomic annotations of TSRs; TTS: transcription termination site, UTR: untranslated region. **(D)** Frequencies of closest annotated features. **(E)** Number of unique genes with at least one associated TSR by gene annotation type. (D, E): snRNA: small nuclear RNA; scRNA: small conditional RNA; snoRNA: small nucleolar RNAs; ncRNA: non-coding RNA; all panels: n=5 paired mTEC samples. Source data for all panels are provided in the Source Data file.

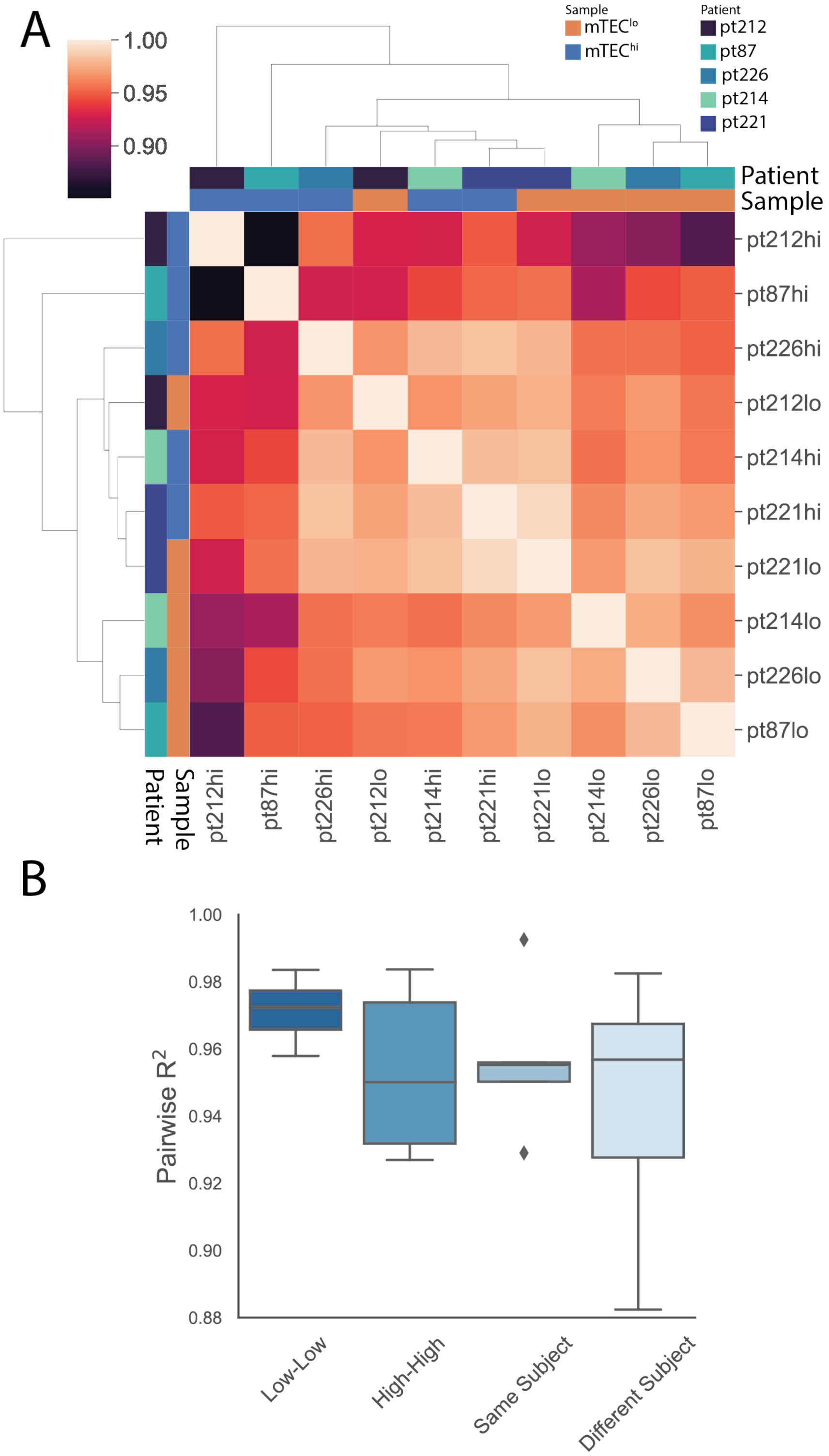

Supplementary Figure 3. Transcription start region (TSR) expression clusters by mTEC^{hi} and mTEC^{lo} samples. **(A)** Clustered heatmap demonstrating pairwise correlation (R^2) between TSR expression in mTEC^{hi} (blue) and mTEC^{lo} (orange) samples. **(B)** Pairwise comparison (Pearson R^2) of TSR expression demonstrates a stronger correlation between paired mTEC^{lo} (Low-Low) or mTEC^{hi} (High-High) samples than for paired mTEC^{hi}-mTEC^{lo} samples taken from one individual (Same Subject); all panels: n=5 paired mTEC samples. Source data for all panels are provided in the Source Data file.

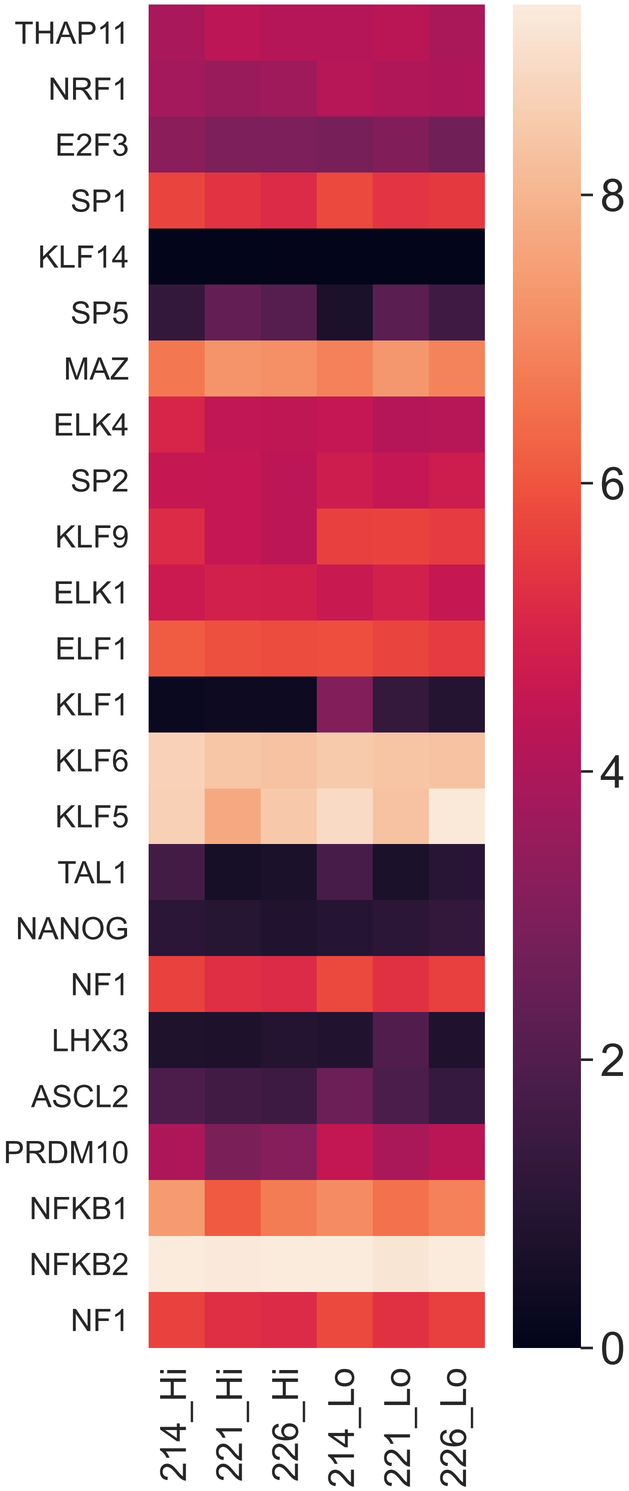

Supplementary Figure 4. Transcription factors with enriched binding motifs are expressed in both mTEC^{hi} and mTEC^{lo} samples. Absolute transcription factor expression levels (color scale) are shown as log_2(transcripts per million) for those transcription factors with enriched motifs in either mTEC population (see Figure 3A); all panels: n=5 paired mTEC samples. Source data are provided in the Source Data file.

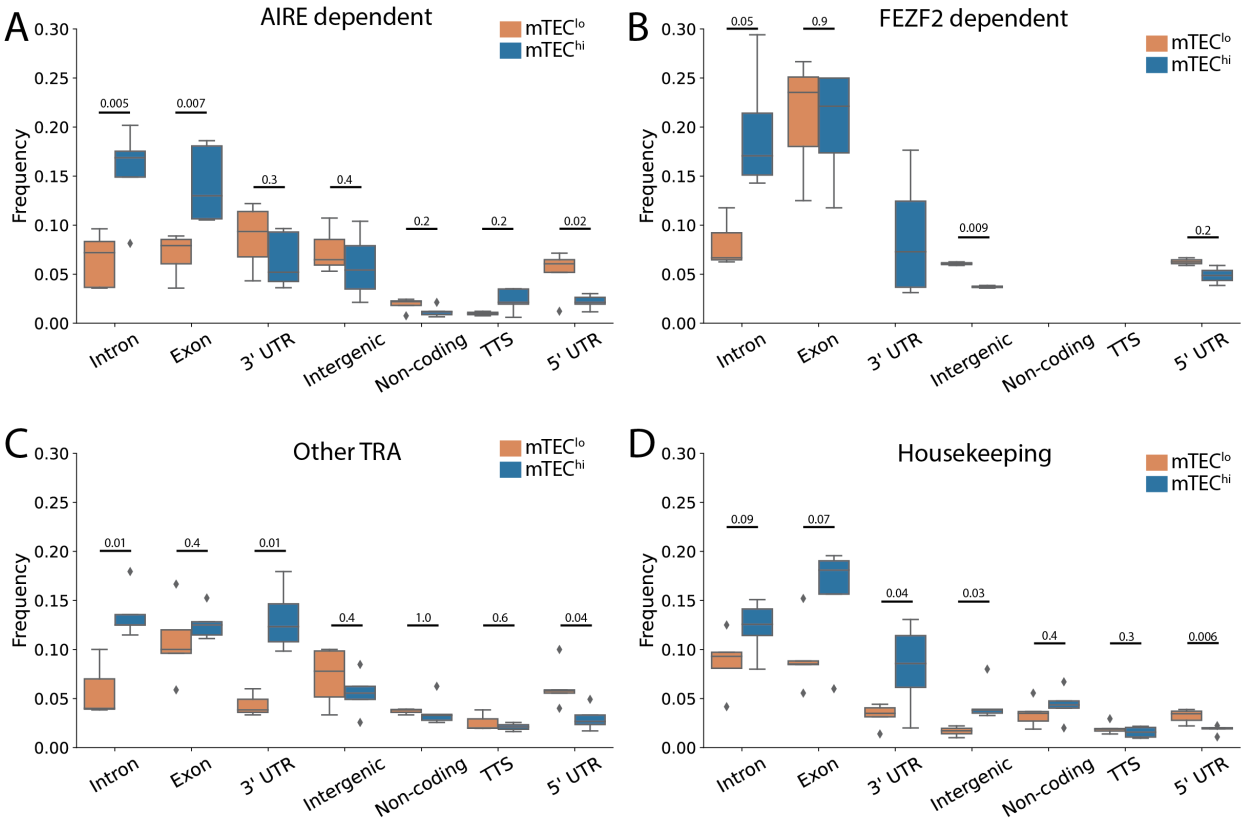

Supplementary Figure 5. mTEC^{hi}- and mTEC^{lo}-specific TSR distributions by gene type. **(A)** Corresponding to AIRE induced and **(B)** FEZF2 induced genes in Figure 3E, the distribution of genomic location annotations are shown for both the mTEC^{hi}- and mTEC^{lo}-specific TSRs. **(C)** Genomic location distributions are additional shown for other TRAs (*i.e.* TRAs that are not known to be induced by AIRE or FEZF2) and **(D)** housekeeping genes. All panels: n=5 paired mTEC samples. Boxplots show median (central line) with interquartile range (IQR, box) and extrema (whiskers at 1.5× IQR). Outliers beyond 1.5× IQR are shown as dots. TTS- transcription termination site; UTR- untranslated region. Values above horizontal bars indicate p-values derived by two-sided paired T test. Source data for all panels are provided in the Source Data file.

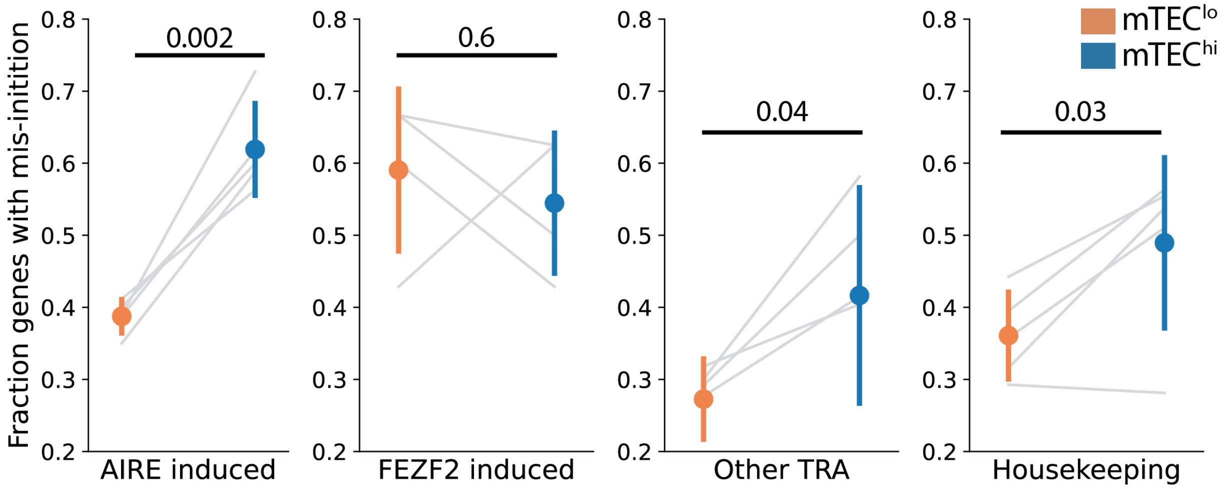

Supplementary Figure 6. Fraction of genes with mis-initiated transcripts. **(A)** A set of genes with similar expression levels was identified according to expression levels in the bulk RNA-sequencing data. We specifically considered those ∼10,000 genes with expression between 10 and 30 transcripts per million (n=9,957, mean ± standard deviation: 16.7 ± 5.4 TPM). The fraction of these genes with at least one mis-initiated TSR (*i.e.* mapping to a genomic location outside of a known promoter region) were calculated for AIRE induced, **(B)** FEZF2 induced, **(C)** other TRAs (*i.e.* TRAs that are not known to be induced by AIRE or FEZF2), and **(D)** housekeeping genes; ll panels: n=5 paired mTEC samples. Values above horizontal bars indicate p-values derived by two-sided paired T test. Source data for all panels are provided in the Source Data file.

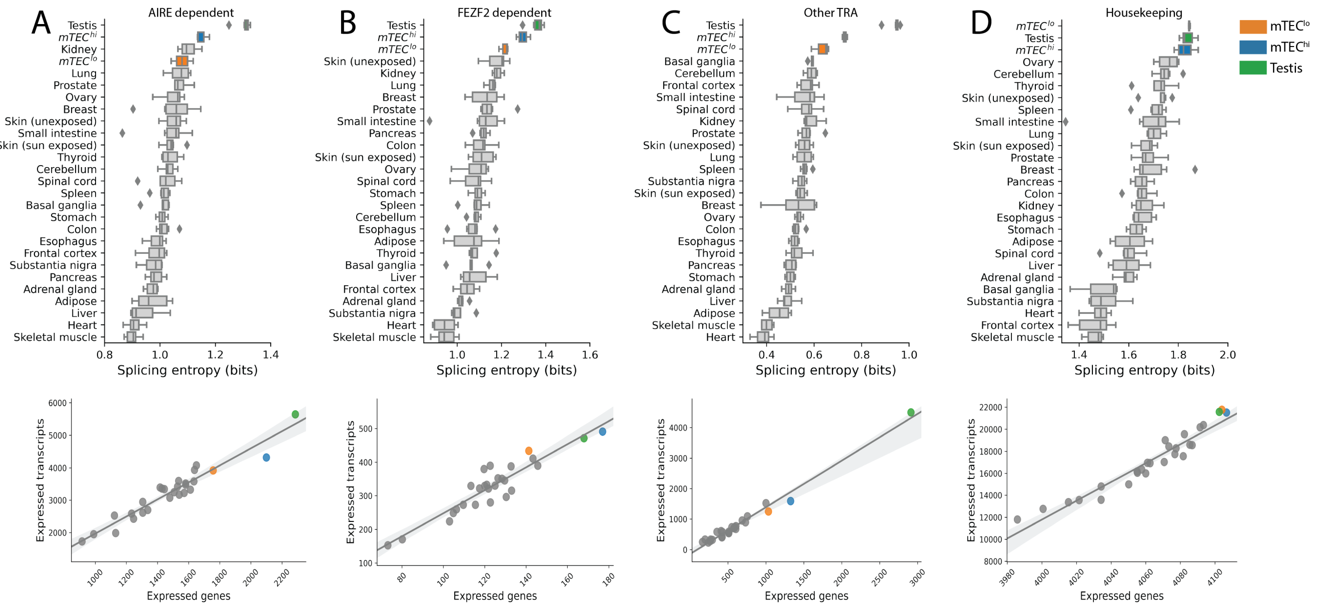

Supplementary Figure 7. Alternative splicing in mTECs by gene type. **(A)** Splicing entropy (upper row) and a linear regression fitting (lower row) the number of expressed transcripts as a function of the number of expressed genes (as in Figure 5A,C, respectively) are shown for AIRE dependent, **(B)** FEZF2 dependent, **(C)** other TRAs (*i.e.* TRAs that are not known to be induced by AIRE or FEZF2), and **(D)** housekeeping genes. For each gene type, the mTEC^{hi} and mTEC^{lo} populations (n=5 paired mTEC samples) are shown alongside 25 peripheral tissue types from GTEx (n=6 samples per tissue). Boxplots show median (central line) with interquartile range (IQR, box) and extrema (whiskers at 1.5× IQR). Outliers beyond 1.5× IQR are shown as dots. Gray shaded area in the linear regression fit marks 95% confidence interval. Source data for all panels are provided in the Source Data file.

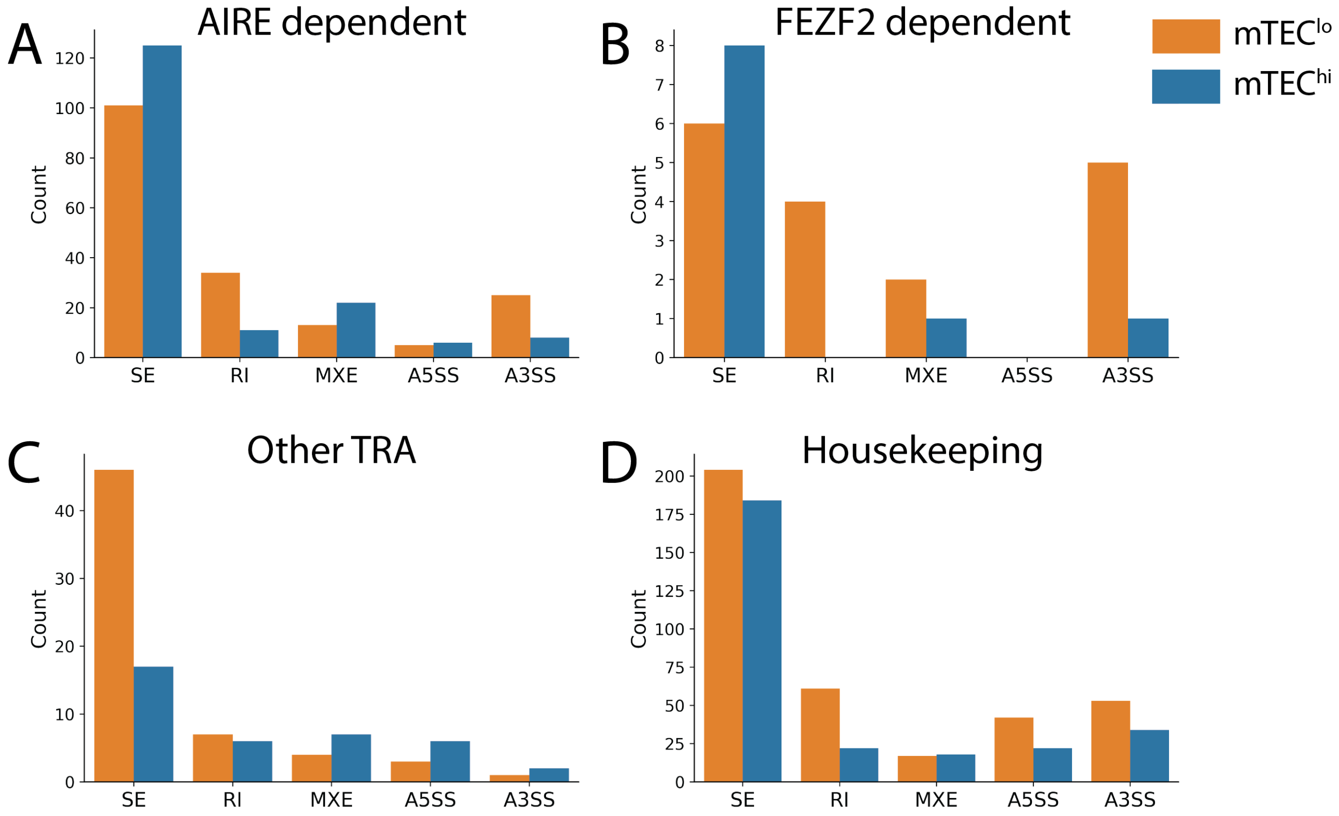

Supplementary Figure 8. Differential splicing by gene type. (**A**) As in Figure 5D, differential splicing between the mTEC^{hi} and mTEC^{lo} populations as predicted by rMATS is shown for AIRE dependent, **(B)** FEZF2 dependent, **(C)** other TRAs (*i.e.* TRAs that are not known to be induced by AIRE or FEZF2), and **(D)** housekeeping genes. Skipped exons (SE), retained introns (RI), alternative 5' and 3' splice sites (A5SS, A3SS), and mutually exclusive exons (MXE). All panels: n=5 paired mTEC samples. Source data for all panels are provided in the Source Data file.

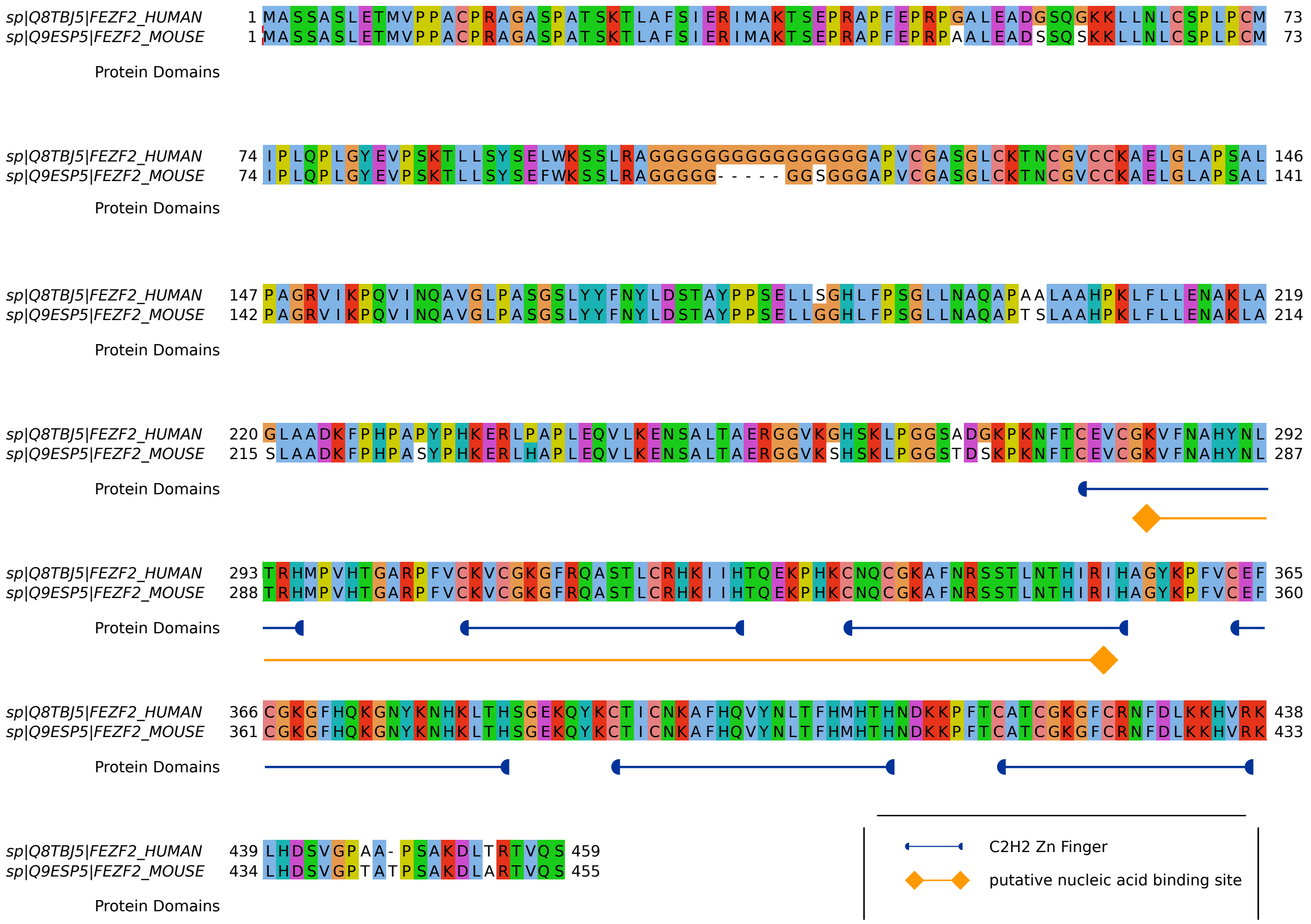

Supplementary Figure 9. Human and murine FEZF2 protein alignment. Clustalx alignment of human and murine FEZF2 protein sequences obtained from uniprot (with respective uniprot IDs). They share 96.48% sequence homology and all protein domains predicted by CD-search[1] are conserved. Both proteins have six Zinc (C2H2 Zn) finger domains forming a predicted nucleic acid binding site.

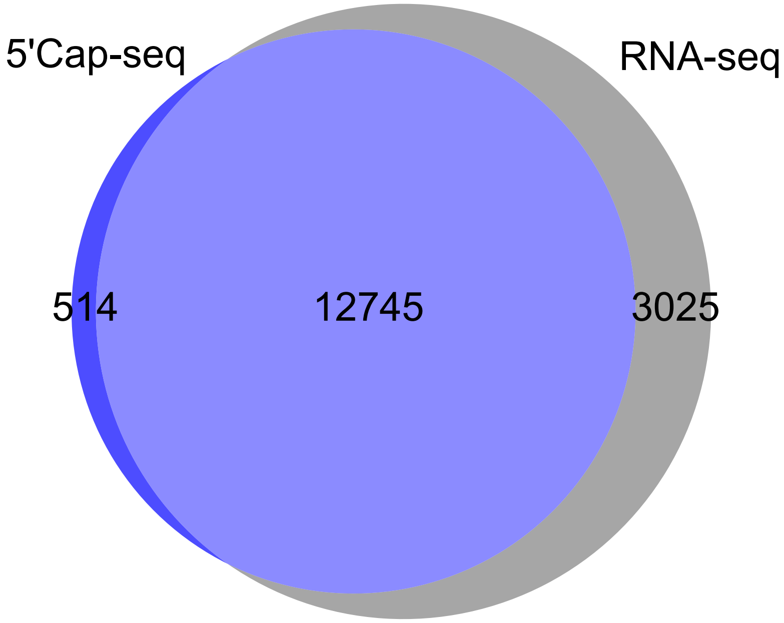

Supplementary Figure 10. Evidence for gene expression in the human mTEC compartment by 5'Cap-seq and RNAseq. Transcription start regions (TSRs) identified by 5'Cap-seq and gene expression detected by conventional RNA-seq, both mapped to ENSEMBL Gene identifiers. Numbers indicate the unique number of protein-coding genes that were identified, with multiple TSRs and transcripts per unique gene possible. Out of all genes with TSR evidence, 96.1% were also detected with RNA-seq. n=5 paired mTEC samples. Source data are provided in the Source Data file.

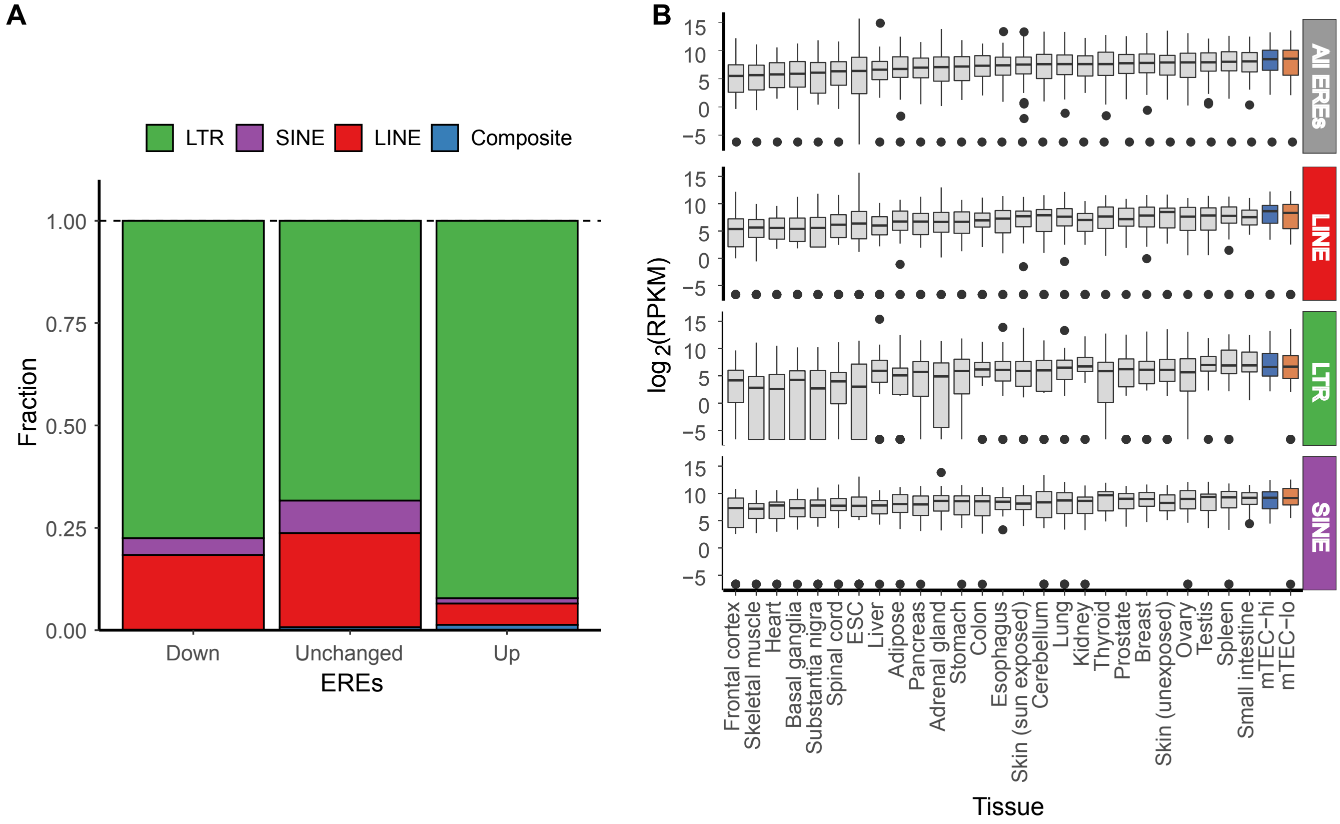

Supplementary Figure 11. Endogenous retroelement (ERE) locus enrichment. **(A)** Expression changes in ERE subfamilies in $mTEC^{hi}$ versus $mTEC^{lo}$ color-coded by class. **(B)** Expression of 108 ereMAPs[2] in mTEC populations, embryonic stem cells (ESCs) and 22 tissues from healthy GTEx samples ordered from lowest to highest median expression. Top (grey facet): overall expression; Bottom (color-coded facets): expression split by ERE classes. Boxplots show median (central line) with interquartile range (IQR, box) and extrema (whiskers at 1.5× IQR). Outliers beyond 1.5× IQR are shown as dots. n=5 paired mTEC samples, n=6 samples per GTEx tissue. Source data for all panels are provided in the Source Data file.

2 Supplementary Tables

Supplementary Table 1. Patient characteristics. Patients underwent surgical repair of non-syndromic congenital cardiac malformations at the indicated age, during which their thymus was (partially) removed.

Patient ID	Age	Sex	5'Cap-seq	RNA-seq
87	4 months	female	yes	no
212	2 weeks	male	yes	no
214	6 days	female	yes	yes
221	4 months	male	yes	Yes
226	9 months	male	yes	yes

Supplementary Table 2. FANTOM5 tissues and accession numbers. Fastq files of tissue samples were downloaded from the DNA Data Bank of Japan at `ftp://ftp.ddbj.nig.ac.jp/ddbj_database/dra/fastq/DRA000/{DRA_sample}/{DRA_experiment_accession}/{DRA_run_accession}.fastq.bz2`, where the expressions in { } are substituted with the respective column entries. Data accessed on June 26, 2021.

Tissue	DRA_sample	DRA_experiment_accession	DRA_run_accession
brain	SAMD00005144	DRX008626	DRR009498
colon	SAMD00005815	DRX008638	DRR009510
esophagus	SAMD00004818	DRX008649	DRR009521
heart	SAMD00005389	DRX008665	DRR009537
kidney	SAMD00005317	DRX008670	DRR009542
liver	SAMD00005542	DRX008674	DRR009546
lung	SAMD00005303	DRX008678	DRR009550
ovary	SAMD00005233	DRX008697	DRR009569
small intestine	SAMD00004876	DRX008724	DRR009596
testis	SAMD00005861	DRX008739	DRR009611
thymus	SAMD00005063	DRX008745	DRR009617
thyroid	SAMD00004739	DRX008747	DRR009619
testis2	SAMD00005187	DRX008740	DRR009612

Supplementary Table 3. GTEx tissues and GUIDs. Bam files for each tissue type were downloaded via the gen3-client using the listed GUIDs. All 25 tissue types were used for the analyses in Figure 5 & Figure 6. Skin (Sun Exposed), Basal Ganglia, and Substantia Nigra were not used in the identification of TRAs or in the analyses in Figure 4.

Tissue	GTEx Samples
Lung	GTEX-QEG5-1126-SM-33HC2, GTEX-T8EM-0326-SM-3DB7F, GTEX-1A8G6-0726-SM-73KV5, GTEX-T2IS-0526-SM-32QP9, GTEX-U3ZN-0626-SM-3DB7U, GTEX-1S82P-0826-SM-EVR4P
Colon	GTEX-1KANA-1926-SM-DHXKF, GTEX-1JKYN-2326-SM-CGQG7, GTEX-ZDTT-1926-SM-5HL5W, GTEX-14BMU-1526-SM-5TDE6, GTEX-131XG-1826-SM-5LZV4, GTEX-ZPCL-1826-SM-57WF1
Stomach	GTEX-1H1DE-1226-SM-9JGHH, GTEX-1K2DA-0826-SM-CGQGD, GTEX-OOBJ-1526-SM-3NB1Q, GTEX-1LGOU-1526-SM-D3LAJ, GTEX-P4PP-1526-SM-3P61M, GTEX-1211K-1426-SM-5FQTF
Prostate	GTEX-1KANA-1026-SM-D3L9L, GTEX-1IDJF-2126-SM-AHZ2S, GTEX-1B8KE-1026-SM-731EQ, GTEX-1K2DA-1226-SM-CGQGH, GTEX-1KXAM-1126-SM-E9TJU, GTEX-132QS-1126-SM-5P9GC
Cerebellum	GTEX-T5JC-2326-SM-32PMR, GTEX-18465-2926-SM-7KFSE, GTEX-1I1GV-3026-SM-CNNR9, GTEX-T2IS-2926-SM-32QPO, GTEX-QDT8-3026-SM-32PKB, GTEX-TSE9-2926-SM-3DB77
Breast	GTEX-1JKYR-0926-SM-CMKGJ, GTEX-1K2DA-1726-SM-CGQGS, GTEX-UTHO-1026-SM-3GAF7, GTEX-15ER7-1626-SM-6PAMZ, GTEX-16NGA-0826-SM-718AF, GTEX-1ICG6-1326-SM-ACKWR
Adipose	GTEX-12C56-1626-SM-5FQUO, GTEX-Q2AH-1726-SM-DIPDV, GTEX-1HSMP-0226-SM-CGQFI, GTEX-T5JW-1726-SM-3GADN, GTEX-U3ZN-2626-SM-3DB7T, GTEX-1ICG6-2626-SM-CGQG1
Spleen	GTEX-1PDJ9-0226-SM-DPRYX, GTEX-1K2DA-0126-SM-CGQGV, GTEX-1R7EU-2026-SM-E9U63, GTEX-ZP4G-0326-SM-4YCEF, GTEX-1LVAN-1026-SM-CNNQZ, GTEX-13PVR-0326-SM-5RQJY
Testis	GTEX-1LBAC-1826-SM-D3L9X, GTEX-1IDJF-2226-SM-AHZ2T, GTEX-1JKYN-1026-SM-CGQG4, GTEX-1399R-1626-SM-5P9GG, GTEX-132QS-1226-SM-5P9GD, GTEX-N7MS-0126-SM-3TW8O
Skeletal Muscle	GTEX-1IDJF-0326-SM-ADEIG, GTEX-1HCVE-0326-SM-CGQEW, GTEX-13N2G-2326-SM-5J1ON, GTEX-15ER7-0226-SM-7KUEU, GTEX-U3ZN-2226-SM-3DB88, GTEX-1J8JJ-0126-SM-D3L93
Adrenal Gland	GTEX-O5YT-1326-SM-3MJGR, GTEX-1K2DA-0226-SM-CGQGW, GTEX-1KXAM-0326-SM-EV7AQ, GTEX-14PKV-0726-SM-686ZF, GTEX-1LVAN-0826-SM-CNNPG, GTEX-Y5V5-1326-SM-4V6G9
Thyroid	GTEX-QEG5-0826-SM-2I5GF, GTEX-1JKYN-1126-SM-CGQG5, GTEX-N7MS-2326-SM-2HMLD, GTEX-PX3G-2626-SM-2I3EG, GTEX-U3ZN-0326-SM-3DB86, GTEX-1IDJU-1626-SM-CJI4B
Esophagus	GTEX-1JKYR-2126-SM-CNNPC, GTEX-1K2DA-0626-SM-CGQGO, GTEX-1H23P-1426-SM-9JGH5, GTEX-1LGOU-0526-SM-D3LAO, GTEX-Y114-0926-SM-4TT7J, GTEX-Y5V5-1626-SM-4VDSG
Skin (not exposed)	GTEX-1IDJF-2026-SM-CL53V, GTEX-1I6K6-1726-SM-CGQFD, GTEX-1AX9J-1326-SM-731BJ, GTEX-15ER7-0626-SM-6PANF, GTEX-ZVT4-2426-SM-5GIEI, GTEX-1F52S-2926-SM-7MKGA
Skin (exposed)	GTEX-1LBAC-0126-SM-DIPEZ, GTEX-1JKYN-1326-SM-CGQG9, GTEX-14PII-0226-SM-6EU1T, GTEX-1LGOU-2026-SM-DIPFF, GTEX-1HCUA-2426-SM-ADEIS, GTEX-1ICG6-2526-SM-CGQFZ
Basal Ganglia	GTEX-R55E-0011-R5A-SM-2TC5N, GTEX-PVOW-0011-R5A-SM-32PL7, GTEX-1HGF4-0011-R5b-SM-CM2ST, GTEX-15ER7-0011-R5b-SM-6M46T, GTEX-13SLX-0011-R5a-SM-5O9BV, GTEX-TSE9-0011-R5A-SM-3DB7J
Small Intestine	GTEX-11P82-0826-SM-5P9GU, GTEX-Y3I4-1326-SM-4TT8Z, GTEX-ZDTT-1726-SM-5HL69, GTEX-1JN76-1126-SM-C1YRL, GTEX-ZLWG-1626-SM-DNZZ1, GTEX-Y5V5-1826-SM-4VDS5
Liver	GTEX-O5YT-0826-SM-3TW8N, GTEX-1399R-1226-SM-5P9GF, GTEX-132NY-0926-SM-5P9G3, GTEX-PX3G-0826-SM-48TZS, GTEX-WYVS-1926-SM-4PQZ2, GTEX-1QCLZ-1226-SM-EAZ49
Spinal Cord	GTEX-T5JC-0011-R9A-SM-32PLV, GTEX-WL46-0011-R9A-SM-3MJFP, GTEX-1GN73-0011-R9a-SM-CKZO5, GTEX-15ER7-0011-R9b-SM-6PALF, GTEX-1GN1U-0011-R9b-SM-CMKFW, GTEX-13PLJ-0011-R9b-SM-5O9DV
Ovary	GTEX-15ER7-2326-SM-7KUN3, GTEX-1LGOU-1026-SM-DHXL4, GTEX-11P81-1526-SM-5P9GS, GTEX-WXYG-1426-SM-4ONCK, GTEX-1ICG6-1526-SM-C1YQT, GTEX-1J8JJ-0826-SM-AHZ3F
Pancreas	GTEX-1JKYR-1026-SM-CGQGI, GTEX-1JKYN-0426-SM-CGQG2, GTEX-1J8Q3-0626-SM-AHZ3V, GTEX-14BMU-0726-SM-73KXS, GTEX-1CAMS-1926-SM-7DUEU, GTEX-1S82P-1026-SM-EAZ4W
Frontal Cortex	GTEX-12WSE-0011-R10b-SM-5P9JV, GTEX-145MG-0011-R10a-SM-5PNWG, GTEX-12WSA-0011-R10b-SM-5P9ET, GTEX-QDT8-0011-R10A-SM-32PKG, GTEX-WWYW-0011-R10A-SM-3NB35, GTEX-13QIC-0011-R10a-SM-5O9C7
Heart	GTEX-XPT6-0126-SM-4B65S, GTEX-17KNJ-0826-SM-793A4, GTEX-OOBJ-0326-SM-33HBO, GTEX-PWCY-0526-SM-5P9HG, GTEX-U3ZN-1426-SM-3DB87, GTEX-OHPL-0326-SM-33HC8
Kidney	GTEX-QLQW-1626-SM-CMKFE, GTEX-12696-0926-SM-5FQTV, GTEX-1MUQO-2526-SM-E9TJN, GTEX-12WSG-0826-SM-5EQ5A, GTEX-13OVI-1126-SM-5KLZF, GTEX-ZYFG-1626-SM-5GZYY
Substantia nigra	GTEX-T5JC-0011-R2A-SM-32PLZ, GTEX-QMR6-0011-R2A-SM-32PKV, GTEX-X4XX-0011-R2A-SM-3P623, GTEX-15ER7-0011-R2a-SM-6M46O, GTEX-QDT8-0011-R2A-SM-32PKQ, GTEX-15DYW-0011-R2b-SM-7KUKY

3 Supplementary Method: Estimating TRAs from GTEx

Tissue-restricted antigens

During T cell development in the thymus, thymocytes encounter more then 85% of all protein, including proteins that are usually expressed in a tissue specific manner. In the following, we use a gene expression data set of about 50k transcripts across 30 tissues (GTEx v8) to estimate tissue specific genes. We use the measure τ as introduced in Yanai 2005 and benchmarked in Kryuchkova-Mostacci 2017 to determine a list of human tissue-restricted antigens:

$$\tau = \frac{\sum_{i=1}^{n}(1-\hat{x}_i)}{n-1}$$

with

$$\hat{x}_i = \frac{x_i}{\max\limits_{1 \le i \le n} x_i}$$

where x_i is the expression of the gene in tissue i and n is the number of tissues.

Setup

```
library(tidyverse)
box::use(tra=./tra)
datadir <- "~/data/tra/gtex"
text_size <- 7
title_size <- 9
```

Download files from GTex: all genes quantified

```
gtex=https://storage.googleapis.com/gtex_analysis_v8/annotations
datadir=~/data/tra/gtex

cd $datadir
wget $gtex/GTEx_Analysis_v8_Annotations_SampleAttributesDS.txt
wget $gtex/GTEx_Analysis_2017-06-05_v8_RNASeQCv1.1.9_gene_tpm.gct.gz
gunzip $gtex/GTEx_Analysis_2017-06-05_v8_RNASeQCv1.1.9_gene_tpm.gct.gz
```

The following description of the Quality Control and gene expression analysis is taken 1:1 from the GTEx website

RNA-seq Alignment

Alignment to the human reference genome hg19/GRCh37 was performed using STAR v2.4.2a, based on the GENCODE v19 annotation. Unaligned reads were kept in the final BAM file. Among multi-mapping reads, one read is flagged as the primary alignment by STAR.

Quantification

Gene-level quantifications: read counts and TPM values were produced with RNA-SeQC v1.1.8 (DeLuca et al., Bioinformatics, 2012), using the following read-level filters:

1. Reads were uniquely mapped (corresponding to a mapping quality of 255 for START BAMs).
2. Reads were aligned in proper pairs.
3. The read alignment distance was <= 6 (i.e., alignments must not contain more than six non-reference bases).
4. Reads were fully contained within exon boundaries. Reads overlapping introns were not counted. These filters were applied using the "-strictMode" flag in RNA-SeQC.

QC and Sample Exclusion Process

1. RNA-seq expression outliers were identified and excluded using a multidimensional extension of the statistic described in (Wright et al., Nat. Genet. 2014). Briefly, for each tissue, read counts from each sample were normalized using size factors calculated with DESeq2 and log-transformed with an offset of 1; genes with a log-transformed value >1 in >10% of samples were selected, and the resulting read counts were centered and unit- normalized. The resulting matrix was then hierarchically clustered (based on average and cosine distance), and a chi2 p-value was calculated based on Mahalanobis distance. Clusters with >=60% samples with Bonferroni-corrected p-values <0.05 were marked as outliers, and their samples were excluded.
2. Samples with <10 million mapped reads were removed.
3. For samples with replicates, the replicate with the greatest number of reads was selected.

Expression analysis

Gene expression values for all samples from a given tissue were normalized using the following procedure:

1. Genes were selected based on expression thresholds of > 0.1 TPM in at least 20% of samples and >=6 reads in at least 20% of samples.
2. Expression values were normalized between samples using TMM as implemented in edgeR (Robinson & Oshlack, Genome Biology, 2010).

3. For each gene, expression values were normalized across samples using an inverse normal transform.

Load gene expression data sets

```
description_attr <- data.table::fread(file.path(datadir,
                                "GTEx_Analysis_v8_Annotations_SampleAttributesDS.txt"),
                                data.table=FALSE) %>%
                    as_tibble

samples_tpm <- data.table::fread(file.path(datadir,
                                "GTEx_Analysis_2017-06-05_v8_RNASeQCv1.1.9_gene_tpm.gct"),
                                data.table=FALSE) %>%
                  as_tibble
```

Format gene expression data sets

Join gene ids, tissue and sample IDs with expression values.

```
tpm_attr <- samples_tpm %>%
    select(Name, Description) %>%
    mutate(ID=gsub("\\..*", "", Name))

write_csv(tpm_attr,
          file.path(datadir,
                    "GTEx_Analysis_2017-06-05_v8_RNASeQCv1.1.9_gene_tpm_attr.csv")
)

tpm_annotated <- samples_tpm %>%
    select(-Description, -Name) %>%
    t %>%
    magrittr::set_colnames(tpm_attr$Name) %>%
    as_tibble %>%
    mutate(SAMPID=colnames(samples_tpm)[-c(1:2)]) %>%
    inner_join(select(description_attr, SAMPID, SMTS), by = "SAMPID")

write_csv(tpm_annotated,
          file.path(datadir,
                    "GTEx_Analysis_2017-06-05_v8_RNASeQCv1.1.9_gene_tpm_gct_annotated.csv")
)
```

Compute the mean expression per gene and per tissue across biological replicates

```
mean_expression <- tpm_annotated %>%
    select(-SAMPID) %>%
    group_by(SMTS) %>%
    summarise_all(mean, na.rm=TRUE)

tissues_cols <- mean_expression$SMTS
mean_expression <- t(mean_expression[,-1])
colnames(mean_expression) <- tissues_cols
saveRDS(mean_expression, "GTEx_Analysis_v8_gene_mean_expression.Rdata")
```

GTex data: only refseq genes expressed in xx tissues

```
gtex_genes <- read_csv(file.path(datadir,"GTEx_Genes.csv"))
colnames(gtex_genes)[1] <- "ID"
gtex_genes <- gtex_genes %>%
    drop_na(ID) %>%
    column_to_rownames("ID") %>%
    t %>%
    as.data.frame %>%
    rownames_to_column("ID") %>%
    mutate(tissue = gsub("(.*)_GTEX.*", "\\1", ID))

gtex_genes_mean <- gtex_genes %>%
    select(-ID) %>%
    group_by(tissue) %>%
    summarize(across(.fns=mean)) %>%
    ungroup %>%
    column_to_rownames("tissue") %>%
    t

gtex_transcripts <- read_csv(file.path(datadir,"GTEx_Transcripts.csv"))
colnames(gtex_transcripts)[1] <- "ID"
gtex_transcripts <- gtex_transcripts  %>%
    drop_na(ID) %>%
    column_to_rownames("ID") %>%
    t %>%
    as.data.frame %>%
    rownames_to_column("ID") %>%
    mutate(tissue = gsub("(.*)_GTEX.*", "\\1", ID))
gtex_transcripts_mean <- gtex_transcripts %>%
    select(-ID) %>%
    group_by(tissue) %>%
    summarize(across(.fns=mean)) %>%
    ungroup %>%
    column_to_rownames("tissue") %>%
    t
```

Compute list of tissue-specific genes

We follow the processing processing procedure described in the Kryuchkova-Mostacci 2017 benchmarking study:

- All genes where the highest gene expression < 1 TPM are set as not expressed;
- count data are log-transformed after addition of 1 to avoid zero counts.

```
filter_expression <-  function(mean_expression,  tpm=1) {

    gtex_max <- apply(mean_expression, 1, tra$fmax)
    mean_expression_filter <- mean_expression[gtex_max > tpm,]
    mean_expression_filter <- mean_expression_filter + 1

    return(mean_expression_filter)
}
```

```r
get_tra <- function(mean_expression_filter, study, tpm=1) {

    gtex <- data.frame(id=gsub("\\..*", "", rownames(mean_expression_filter)),
                       full=rownames(mean_expression_filter),
                       tau=apply(log2(mean_expression_filter), 1, tra$tau),
                       study=study,
                       stringsAsFactors = FALSE)

    write.table(select(gtex, -study),
            file=file.path(datadir, paste0(study, "_tau.csv")),
            sep=",", col.names=TRUE, row.names=FALSE, quote=FALSE)

    return(gtex)
}

gtex_genes_filter <- filter_expression(gtex_genes_mean)
gtex_transcripts_filter <- filter_expression(gtex_transcripts_mean)
gtex_all_filter <- filter_expression(gtex_all_mean)

gtex_genes_tau <- get_tra(gtex_genes_filter, "gtex_genes")
gtex_transcripts_tau <- get_tra(gtex_transcripts_filter , "gtex_transcripts")
gtex_all_tau <- get_tra(gtex_all_filter, "gtex_genes_all")

gtex_combine <- rbind(gtex_genes_tau,gtex_transcripts_tau, gtex_all_tau)
p <- ggplot(gtex_combine, aes(x=tau))
p + geom_density(aes(color=study)) +
    scale_color_brewer(type='qual', palette = "Set1") +
    cowplot::theme_cowplot() +
    theme(axis.text = element_text(size=text_size),
          axis.title = element_text(size=title_size))
```

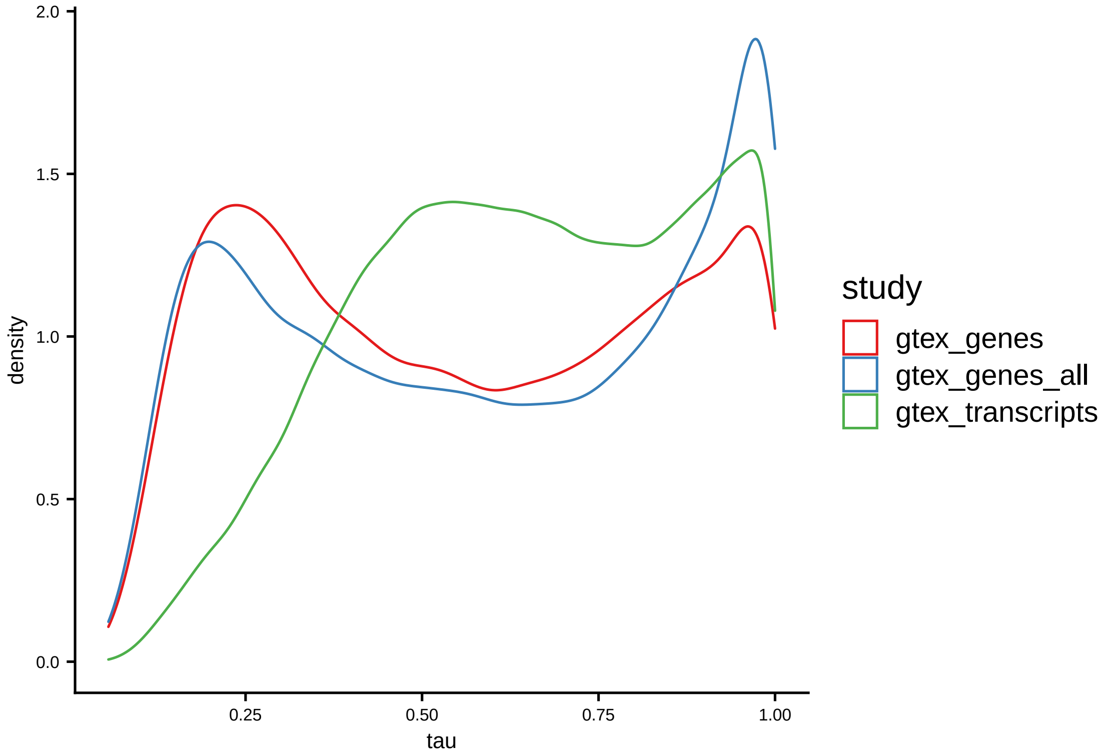


```
tt <- tibble(GTEx = c("All GTEx Genes", "RefSeq Genes", "RefSeq Transcripts"),
       total= c(nrow(gtex_all_mean), nrow(gtex_genes_mean), nrow(gtex_transcripts_mean)),
       expressed = c(nrow(gtex_all_tau),
                            nrow(gtex_genes_tau),
                            nrow(gtex_transcripts_tau)),
       tra_0.7 = c(sum(gtex_all_tau$tau >= 0.7),
                        sum(gtex_genes_tau$tau >= 0.7),
                        sum(gtex_transcripts_tau$tau >= 0.7)),
       tra_0.8 = c(sum(gtex_all_tau$tau >= 0.8),
                        sum(gtex_genes_tau$tau >= 0.8),
                        sum(gtex_transcripts_tau$tau >= 0.8))
)
kableExtra::kable(tt, format="latex",
                  col.names = c("GTEx", "Total", "Expressed: TPM $\\ge$ 1",
                              "TRA: $\\tau \\ge$ 0.7",
                              "TRA: $\\tau \\ge$ 0.8"))
```

GTEx	Total	Expressed: TPM $\ge$ 1	TRA: $\tau \ge$ 0.7	TRA: $\tau \ge$ 0.8
All GTEx Genes	56200	32347	13413	10687
RefSeq Genes	40481	26131	9587	7083
RefSeq Transcripts	194360	118256	50875	35599

Find tissue-specificity of tissue-specific genes

- use binarizing approach described by Yanai 2005

```
find_tissue_spec <- function(mean_expression_filter, feature_tau, study,
                             tauthr=0.8) {
    feature_tau <- feature_tau[feature_tau$tau > tauthr,]
    mean_expression_tra <- mean_expression_filter %>%
        as.data.frame %>%
        rownames_to_column("full") %>%
        filter(full %in% feature_tau$full) %>%
        column_to_rownames("full") %>%
        as.matrix

    expression_tra_tissues <- apply(mean_expression_tra, 1,
                                    tra$find_tra_tissues) %>%
        t %>%
        as.data.frame %>%
        rownames_to_column("full") %>%
        left_join(feature_tau) %>%
        select(-full, -study) %>%
        select(id, tau, everything()) %>%
        as_tibble()

    write.table(expression_tra_tissues,
            file=file.path(datadir, paste0(study, "_binarized_expression.csv")),
            sep=",", col.names=TRUE, row.names=FALSE, quote=FALSE)

    return(expression_tra_tissues)
}

gtex_genes_tissues <- find_tissue_spec(gtex_genes_filter, gtex_genes_tau,
                                       "gtex_genes")
gtex_transcripts_tissues <- find_tissue_spec(gtex_transcripts_filter, gtex_transcripts_tau,
                                       "gtex_transcripts")
gtex_genes_all_tissues <- find_tissue_spec(gtex_all_filter, gtex_all_tau,
                                       "gtex_genes_all")
```

- visualise distribution of TRAs per tissue

```
visualise_tra_per_tissue <- function(expression_tra_tissues) {
    tras_per_tissue <- expression_tra_tissues %>%
        mutate(thr=case_when(tau < 0.8 ~ "> 0.7",
                             tau >= 0.8 & tau < 0.9 ~ "> 0.8",
                             tau >= 0.9 ~ "> 0.9")) %>%
        pivot_longer(-c(id, tau, thr),
                     names_to="tissue", values_to="status") %>%
        filter(status != 0) %>%
        group_by(tissue, thr) %>%
        summarise(tras = n(), .groups='drop') %>%
        arrange(tras) %>%
        mutate(tissue = fct_inorder(tissue))

    p_all_tissues <- ggplot(tras_per_tissue) +
        geom_bar(aes(x=tissue, y=tras, fill=thr), stat='identity') +
        scale_fill_manual(values=c('#66c2a5','#fc8d62','#8da0cb'), guide=FALSE) +
        labs(x="GTEx tissues",
             y="Number of TRAs",
```

```r
             fill="Tau threshold") +
        cowplot::theme_cowplot() +
        theme(axis.text.x = element_blank(),
              axis.title.x = element_blank(),
              axis.ticks.x = element_blank(),
              axis.text.y = element_text(size=text_size),
              axis.title.y = element_text(size=title_size))

    p_tissues_no_testes <- ggplot(filter(tras_per_tissue, tissue != "Testis")) +
        geom_bar(aes(x=tissue, y=tras, fill=thr), stat='identity') +
        scale_fill_manual(values=c('#66c2a5','#fc8d62','#8da0cb')) +
        labs(x="GTEx tissues",
             y="Number of TRAs",
             fill="Tau threshold") +
        cowplot::theme_cowplot() +
        theme(axis.text.x = element_text(angle=45, hjust = 1, vjust = 1),
              axis.text = element_text(size=text_size),
              axis.title = element_text(size=title_size),
              legend.position = "bottom")
    cowplot::plot_grid(p_all_tissues, p_tissues_no_testes,
                       nrow=2,
                       align="v",
                       rel_heights = c(1, 2.5),
                       axis="lr"
                       )
}
gtex_genes_tra_per_tissue <- visualise_tra_per_tissue(gtex_genes_tissues)
gtex_transcripts_tra_per_tissue <- visualise_tra_per_tissue(gtex_transcripts_tissues)
gtex_all_tra_per_tissue <- visualise_tra_per_tissue(gtex_genes_all_tissues)

gtex_genes_tra_per_tissue
```

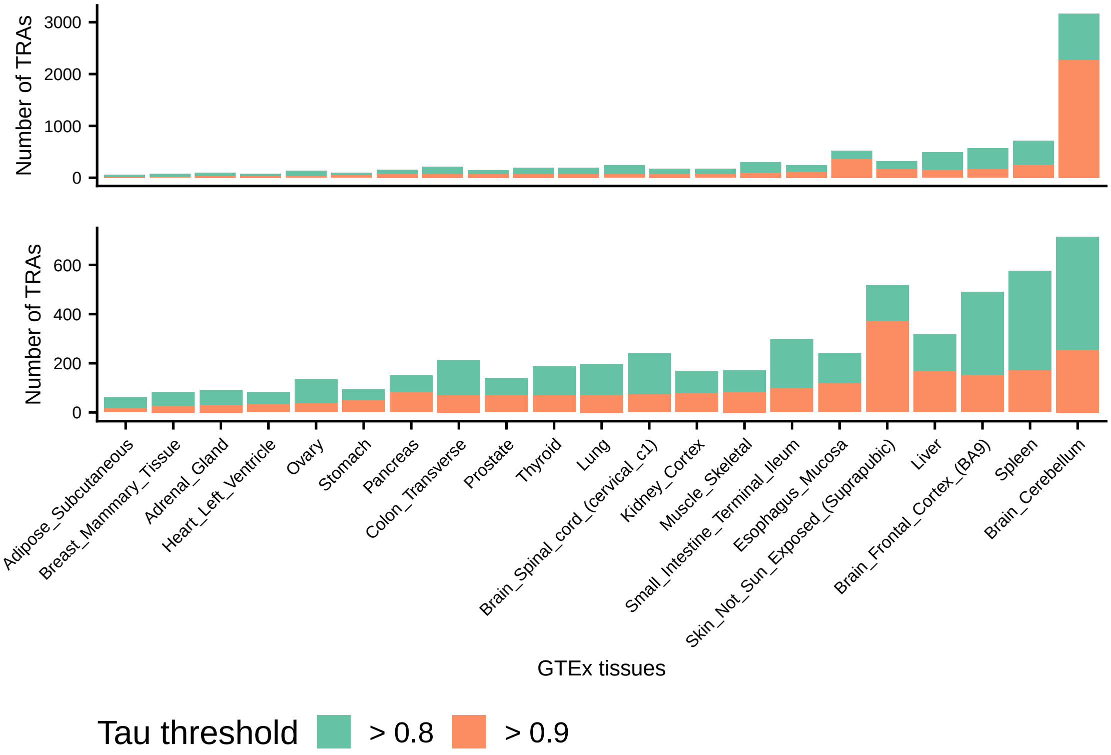


```
gtex_transcripts_tra_per_tissue
```

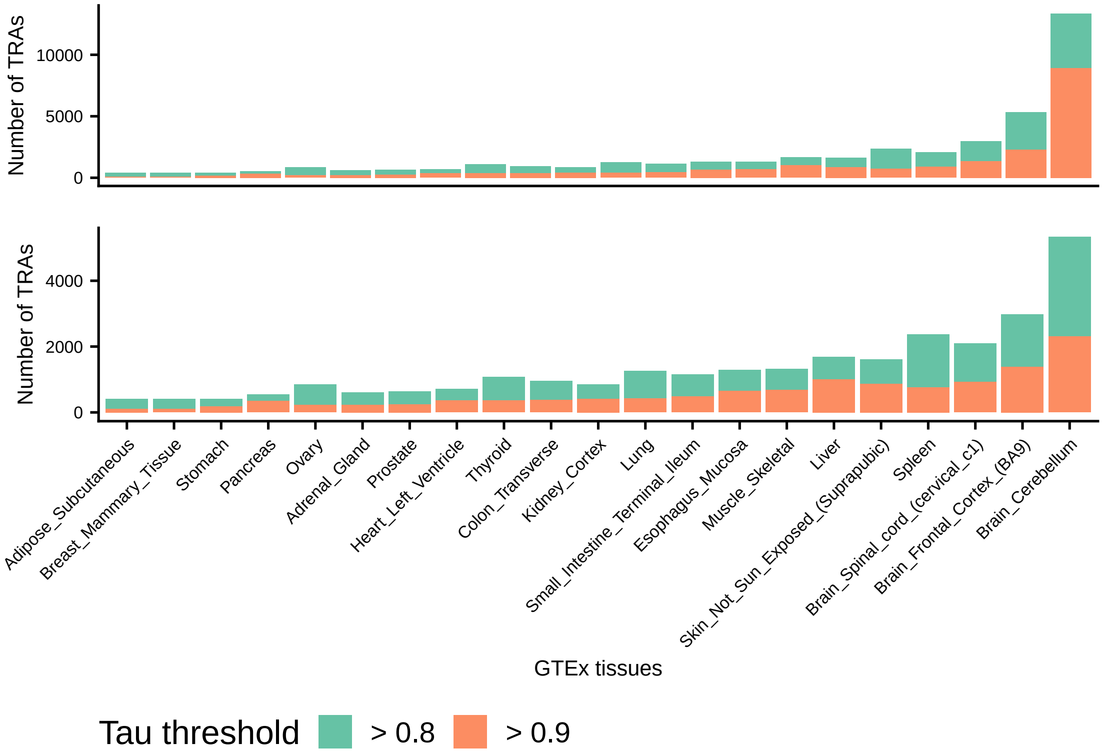


```
gtex_all_tra_per_tissue
```

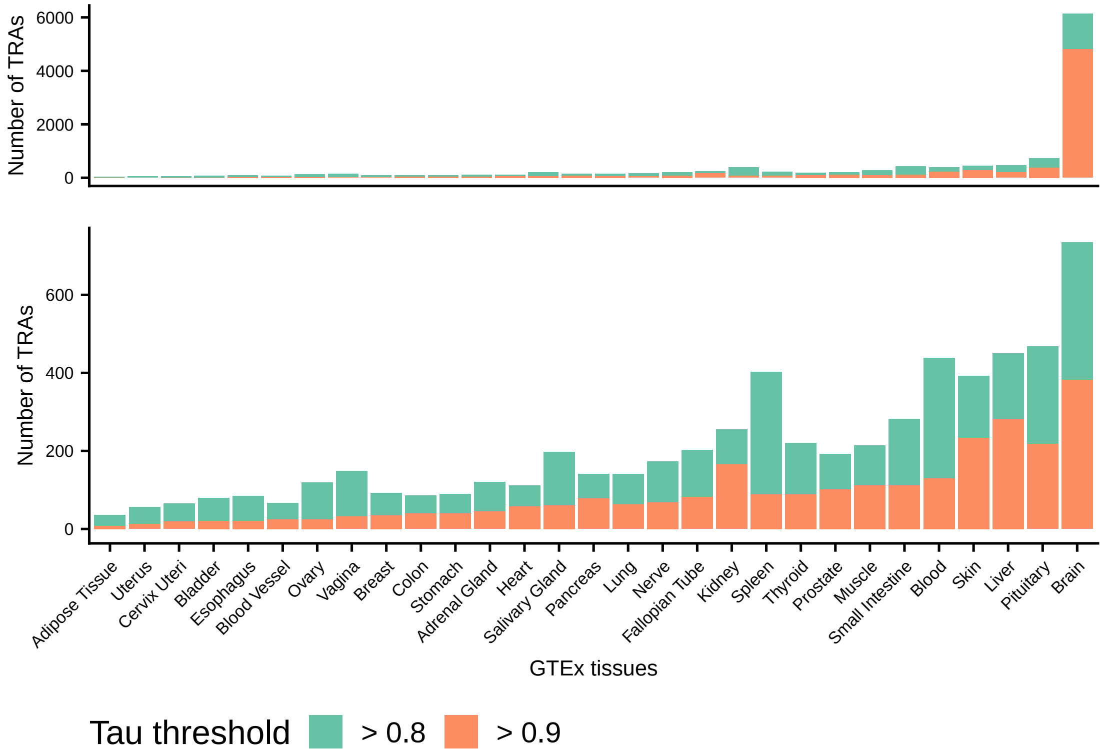

- visualize how many tissues a given TRA is assigned
- visualize relationship between tau and number of tissues expressing TRA

```
visualise_tissue_per_tra <- function(expression_tra_tissues) {
    tissue_per_tra <- expression_tra_tissues %>%
        mutate(thr=case_when(tau < 0.8 ~ "> 0.7",
                             tau >= 0.8 & tau < 0.9 ~ "> 0.8",
                             tau >= 0.9 ~ "> 0.9")) %>%
        pivot_longer(-c(id, tau, thr), names_to="tissue", values_to="status") %>%
        filter(status != 0) %>%
        group_by(id, tau, thr) %>%
        summarise(tissues = n(), .groups='drop') %>%
        arrange(tissues) %>%
        mutate(id = fct_inorder(id))

    p_all_tras <-  ggplot(tissue_per_tra) +
        geom_bar(aes(tissues, fill=thr)) +
        scale_fill_manual(values=c('#66c2a5','#fc8d62','#8da0cb')) +
        scale_x_continuous( breaks=1:6)+
        labs(x="Number of GTEx tissues",
             y="Number of TRAs",
             fill="Tau threshold") +
        cowplot::theme_cowplot() +
        theme(axis.text = element_text(size=text_size),
              axis.title = element_text(size=title_size))

    p_tau_versus_tissue <-  ggplot(tissue_per_tra) +
        geom_boxplot(aes(x=as.factor(tissues), y=tau, color=thr)) +
```

```
        scale_color_manual(values=c('#66c2a5','#fc8d62','#8da0cb')) +
        labs(x="Number of GTEx tissues",
             y="Tau",
             color="Tau threshold") +
        cowplot::theme_cowplot() +
        theme(axis.text = element_text(size=text_size),
              axis.title = element_text(size=title_size))

    cowplot::plot_grid(p_all_tras,p_tau_versus_tissue,
                       nrow=2,
                       align="v",
                       axis="lr")
}

gtex_genes_tissue_per_tra <- visualise_tissue_per_tra(gtex_genes_tissues)
gtex_transcripts_tissue_per_tra  <- visualise_tissue_per_tra(gtex_transcripts_tissues)
gtex_all_tissue_per_tra  <- visualise_tissue_per_tra(gtex_genes_all_tissues)

gtex_genes_tissue_per_tra
```

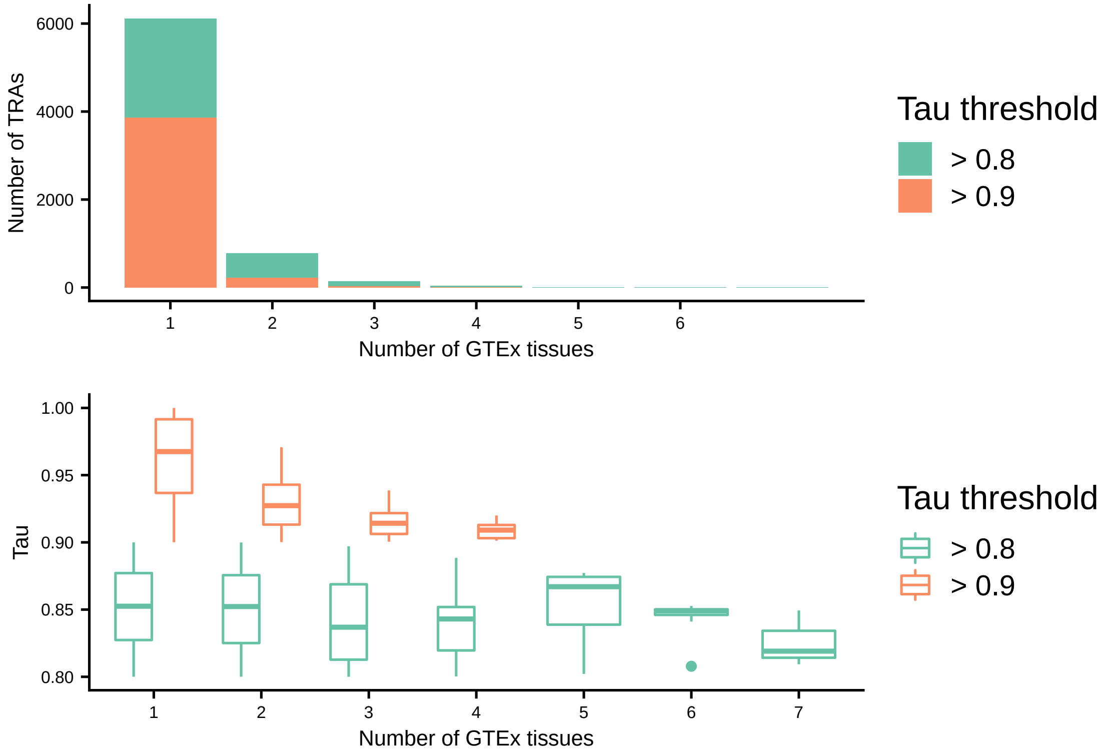


```
gtex_transcripts_tissue_per_tra
```

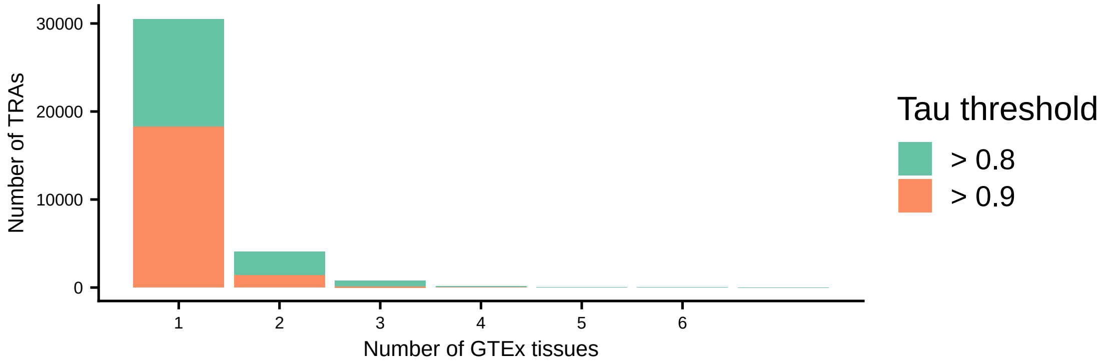

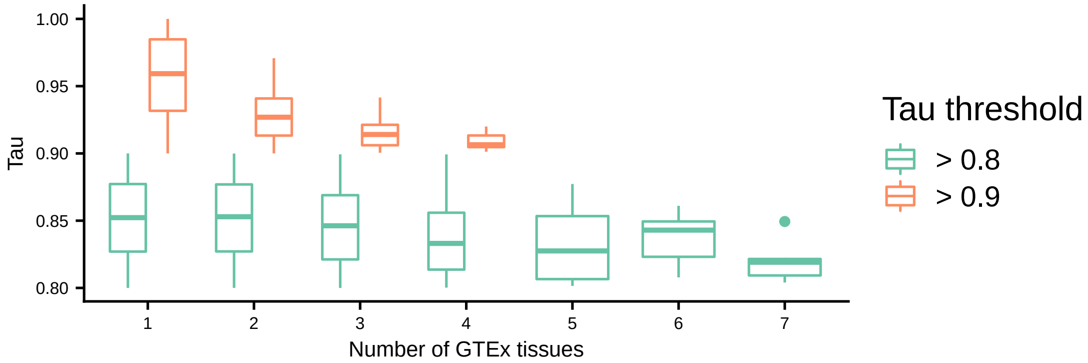


```
gtex_all_tissue_per_tra
```

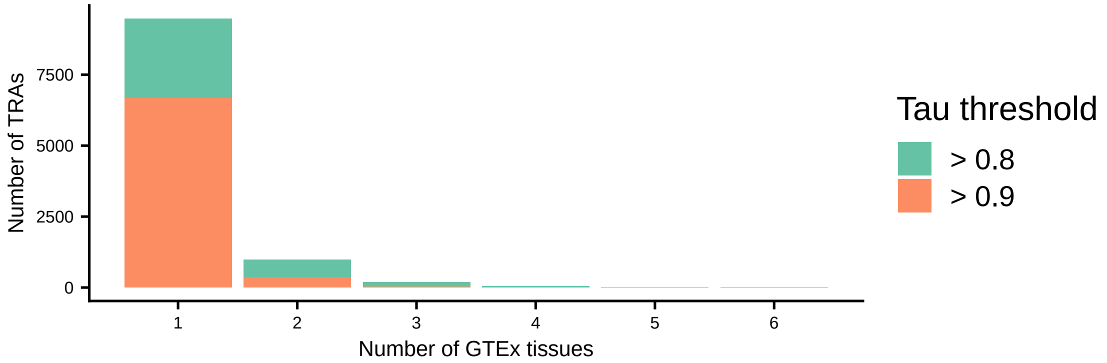

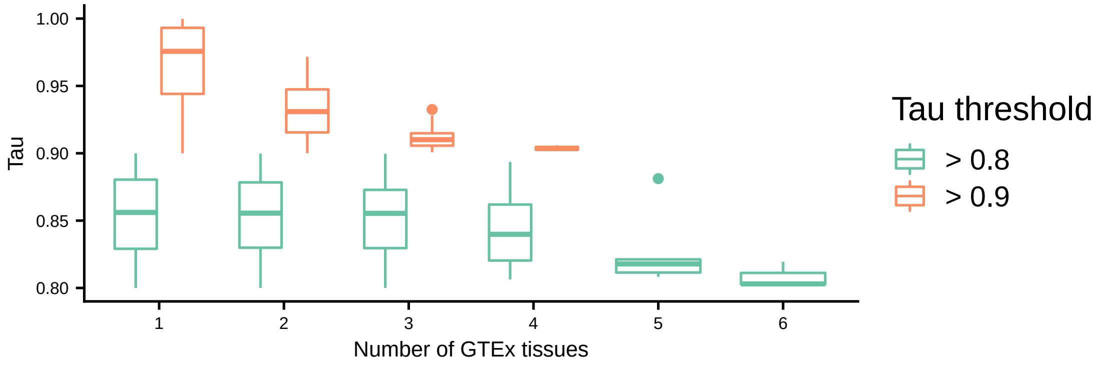

Compare to Fagerberg (2014) data

- Data set derived from Fagerberg (2014) Supplementary data;
- formatting in analogy to formating in Mostacci (2014);
- formatting for zero counts adjusted to adding 1 to each count value instead of only setting 0 counts to 1.

```
fb_file<- "~/data/public/2016_bioinformatics_mostacci/2014_MCP_Fagerberg.xlsx"
orgExpression <- readxl::read_xlsx(fb_file)
colnames(orgExpression)[1] <- "Ensembl.Gene.ID"

orgExpression <-
    orgExpression[regexpr("ENS", orgExpression$Ensembl.Gene.ID) > 0 |
                      regexpr("FBgn", orgExpression$Ensembl.Gene.ID) > 0 |
                      regexpr("PPAG", orgExpression$Ensembl.Gene.ID) > 0, ]
orgExpression <- na.omit(orgExpression[,-29])
x <- orgExpression[,-1] + 1
orgExpression[, -1] <- log2(x)
fagerberg_max <- apply(orgExpression[,-1], 1, tra$fmax)
orgExpression <- orgExpression[fagerberg_max > log2(1),]
fagerberg <- data.frame(id=orgExpression$Ensembl.Gene.ID,
                        full=orgExpression$Ensembl.Gene.ID,
                        tau=apply(orgExpression[,-1], 1, tra$tau),
                        study='fagerberg',
                        stringsAsFactors = FALSE)

fagerberg <- fagerberg %>%
```

```
    left_join(tpm_attr, by = c("id" = "ID")) %>%
    select(Description, everything()) %>%
    select(-Name) %>%
    rename(Gene=Description)

p <- ggplot(fagerberg, aes(x=tau))
p + geom_density(color="#1b9e77") +
    cowplot::theme_cowplot() +
    theme(axis.text = element_text(size=text_size),
          axis.title = element_text(size=title_size))
```

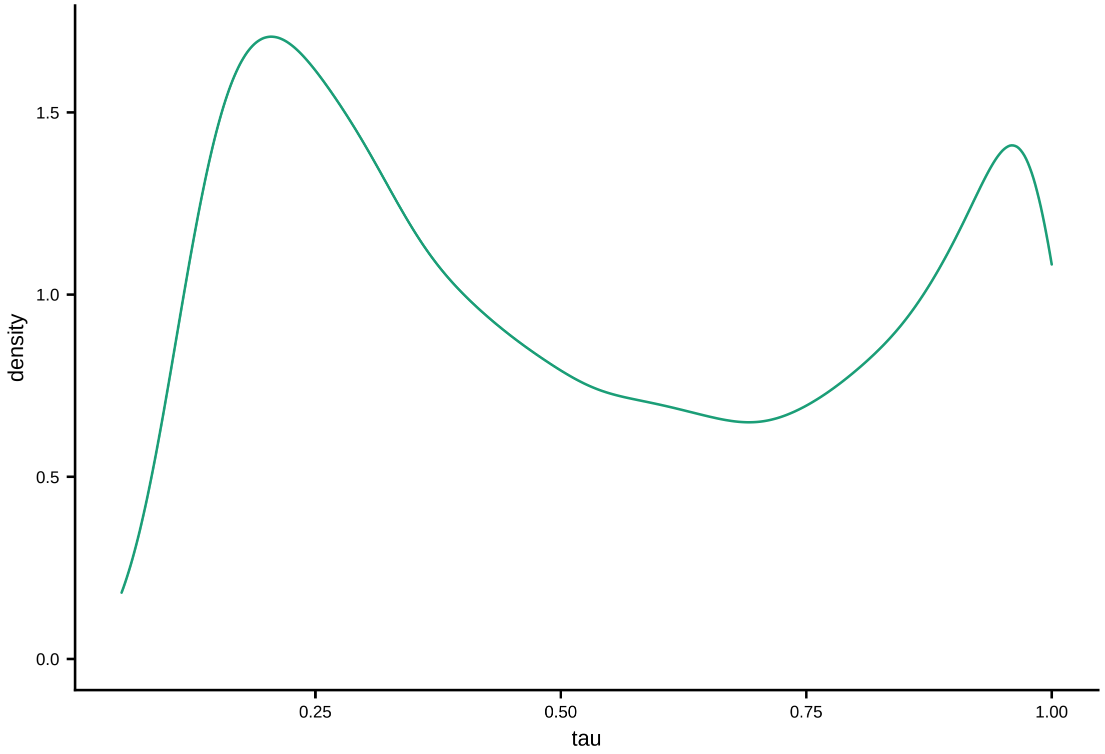

Compare the results of GTEx and Fagerberg tissue specificity

- Fagerberg 19881 genes;
- GTEx (all genes: 32347, refseq only genes 26131);
- Find common genes and compare distribution of tau;
- check overlap of TRA sets

```
all_ids <-  c(fagerberg$id, gtex_all_tau$id, gtex_genes_tau$id)
common <-  table(all_ids)[table(all_ids) == 3]

fagerberg_common <- fagerberg[fagerberg$id %in% names(common),]
gtex_all_genes_common <- gtex_all_tau[gtex_all_tau$id %in% names(common),]
gtex_genes_common <- gtex_genes_tau[gtex_genes_tau$id %in% names(common),]

combined <- rbind(fagerberg_common[, -1], gtex_all_genes_common, gtex_genes_common)
```

```r
p <- ggplot(combined, aes(x=tau, color=study))
p + geom_density() +
    scale_color_manual(values=c("#1b9e77", "#e41a1c", "#377eb8"))+
    cowplot::theme_cowplot() +
    theme(axis.text = element_text(size=text_size),
          axis.title = element_text(size=title_size),
          legend.position = 'bottom')
```

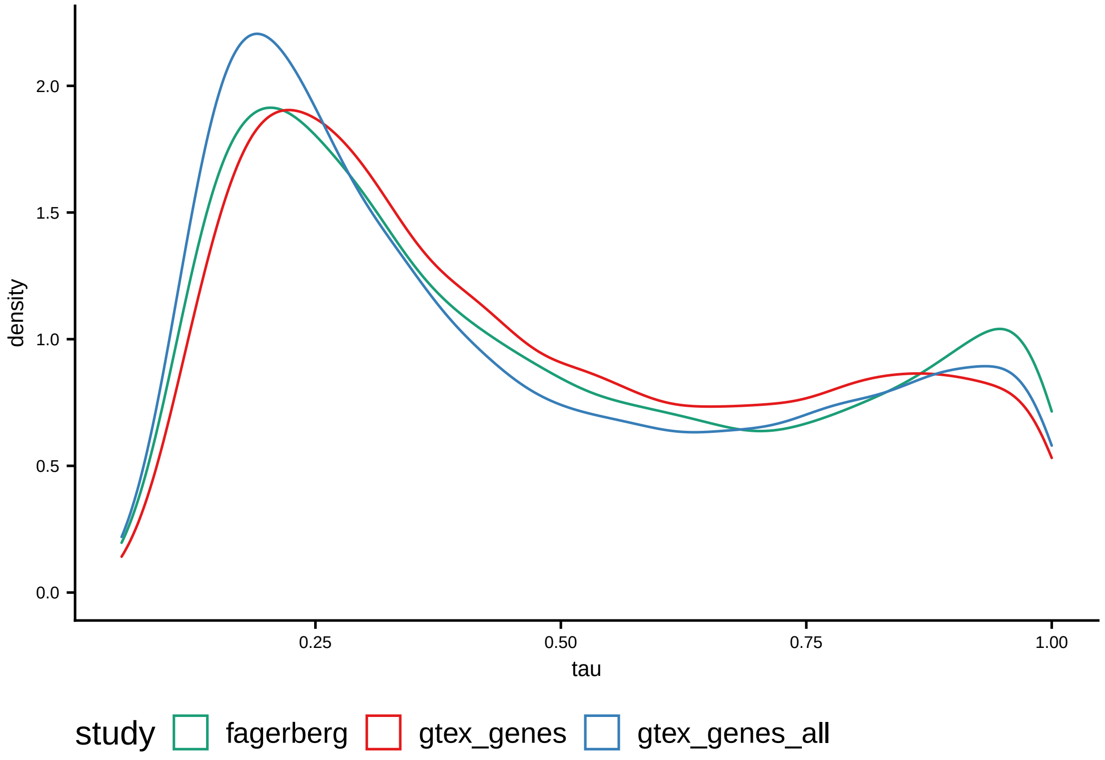


```r
gtex_all_genes_tissue_specific <- gtex_all_genes_common[gtex_all_genes_common$tau > 0.8,]
gtex_genes_tissue_specific <- gtex_genes_common[gtex_genes_common$tau > 0.8,]
fagerberg_tissue_specific <- fagerberg_common[fagerberg_common$tau > 0.8,]

gtex_all_genes_pct <- sum(gtex_all_genes_tissue_specific$id %in% fagerberg_tissue_specific$id)/
    nrow(gtex_all_genes_tissue_specific)

gtex_genes_pct <- sum(gtex_genes_tissue_specific$id %in% fagerberg_tissue_specific$id)/
    nrow(gtex_genes_tissue_specific)

fagerberg_all_genes_pct <- sum(gtex_all_genes_tissue_specific$id %in%
                         fagerberg_tissue_specific$id)/
    nrow(fagerberg_tissue_specific)

fagerberg_genes_pct <- sum(gtex_genes_tissue_specific$id %in%
                         fagerberg_tissue_specific$id)/
    nrow(fagerberg_tissue_specific)
```

There are 17447 common genes in the two GTEx (all genes, refseq genes) and Fagerberg dataset. Of those,

there are 3202 and 3127 genes with $\tau > 0.8$ in the GTEx all genes and refseq genes datasets, respectively, ie genes we consider as TRA, and 3565 TRAs in the Fagerberg dataset. The overlap between these GTEx and Fagerberg datasets are 0.91 and 0.82 for all genes in GTEx and 0.9 and 0.79 for refseq only genes.

References

1. Marchler-Bauer, A. *et al.* CDD: A Conserved Domain Database for the functional annotation of proteins. *Nucleic Acids Research* **39**, 225–229 (2011).
2. Larouche, J. D. *et al.* Widespread and tissue-specific expression of endogenous retroelements in human somatic tissues. *Genome Medicine* **12**, 1–16 (2020).
