# Supplementary material for: Transcriptomic diversity in human medullary thymic epithelial cells
Source: Nat Commun. 2022 Aug 2;13:4296. doi: 10.1038/s41467-022-31750-1 (PMC9345899; doi:10.1038/s41467-022-31750-1)
Supplement: Supplementary file 3 — Reporting Summary [file 41467_2022_31750_MOESM3_ESM.pdf]

nature portfolio

Corresponding author(s): Hannah Meyer

Last updated by author(s): 06-15-2022

Reporting Summary

Nature Portfolio wishes to improve the reproducibility of the work that we publish. This form provides structure for consistency and transparency in reporting. For further information on Nature Portfolio policies, see our Editorial Policies and the Editorial Policy Checklist.

Statistics

For all statistical analyses, confirm that the following items are present in the figure legend, table legend, main text, or Methods section.

n/a	Confirmed	
☐	☒	The exact sample size (*n*) for each experimental group/condition, given as a discrete number and unit of measurement
☐	☒	A statement on whether measurements were taken from distinct samples or whether the same sample was measured repeatedly
☐	☒	The statistical test(s) used AND whether they are one- or two-sided<br>*Only common tests should be described solely by name; describe more complex techniques in the Methods section.*
☐	☒	A description of all covariates tested
☐	☒	A description of any assumptions or corrections, such as tests of normality and adjustment for multiple comparisons
☐	☒	A full description of the statistical parameters including central tendency (e.g. means) or other basic estimates (e.g. regression coefficient) AND variation (e.g. standard deviation) or associated estimates of uncertainty (e.g. confidence intervals)
☐	☒	For null hypothesis testing, the test statistic (e.g. *F*, *t*, *r*) with confidence intervals, effect sizes, degrees of freedom and *P* value noted<br>*Give P values as exact values whenever suitable.*
☒	☐	For Bayesian analysis, information on the choice of priors and Markov chain Monte Carlo settings
☒	☐	For hierarchical and complex designs, identification of the appropriate level for tests and full reporting of outcomes
☐	☒	Estimates of effect sizes (e.g. Cohen's *d*, Pearson's *r*), indicating how they were calculated

Our web collection on statistics for biologists contains articles on many of the points above.

Software and code

Policy information about availability of computer code

Data collection	BD FACSDiva Software (v8.2) and FlowJo -(v7.6) for cell sorting
Data analysis	All analysis in R were conducted with R version ≥ 4.0.3, python version ≥ 3.8. All analyses in this study can be found here:https://github.com/meyer-lab-cshl/transcriptomic-diversity-human-mTECs.<br>Python: python (v3.9.6), IPython (v7.26.0), scipy (v1.7.1), seaborn (v0.11.1), matplotlib (v3.4.2), matplotlib_venn (v0.11.6), numpy (v1.21.1), pandas (v1.3.1), sklearn (v0.24.2), statsmodels (v0.12.2)<br><br>R: R (v4.0.3), CAGEr (v1.32), limma (v3.46), Sleuth (v0.30.0), biomaRt (v2.46.3), rMATS (v4.1.1), DESeq2 (v1.30.1), ngs.plot.r (v2.61), Gviz (v1.34.1)<br><br>ssequence analysis: umi_tools (v1.1), fastq_screen (v0.14.0), STAR (v2.7.2b), samtools (v1.11), picard (v2.18.20), multiqc (v1.9), paraclu (v9), bbedops (v2.4.38), bedtools (v2.29.2), HOMER (v4.11.1), sambamba (v0.8), fastp (v0.11.8), Kallisto (v0.4.6), clustal omega (v1.2.4), FastQc (v0.11.8), TEtranscripts (v2.2.1), TElocal (v1.1.1), SalmonTE (v0.4), LIONS (no version specified; github download July 13, 2021), fastq-dump (v2.5)<br><br>CD-Search - https://www.ncbi.nlm.nih.gov/Structure/cdd/wrpsb.cgi

For manuscripts utilizing custom algorithms or software that are central to the research but not yet described in published literature, software must be made available to editors and reviewers. We strongly encourage code deposition in a community repository (e.g. GitHub). See the Nature Portfolio guidelines for submitting code & software for further information.

Data

Policy information about availability of data

All manuscripts must include a data availability statement. This statement should provide the following information, where applicable:

- Accession codes, unique identifiers, or web links for publicly available datasets
- A description of any restrictions on data availability
- For clinical datasets or third party data, please ensure that the statement adheres to our policy

All data generated in this study are available at the Gene Expression Omnibus, accession number: GSE201720. An interactive interface to explore the data is available at http://transcriptomediversity.cshl.edu/ (implemented using InteractiveComplexHeatmap - v2.9.4 and ShinyDashboard - v0.7.1).

Additional data used in this study is available at the following sources:
* ChIP-seq data for H3K4me3 and H3K27me3in mTEChi cells derived from 4 week old C57BL/6 mice was obtained from the SRA (SRP033578, runs SRR1045003-SRR1045008)
* embryonic stem cell RNA-seq data from was downloaded from SRA (SRR488684 and SRR488685)
* raw gene expression bam files for 25 tissues (Adipose Subcutaneous, Adrenal Gland, Brain Basal Ganglia, Brain Cerebellum, Brain Frontal Cortex (BA9), Brain Spinal cord (cervical c1), Breast Mammary Tissue, Colon Transverse, Esophagus Mucosa, Heart Left Ventricle, Kidney Cortex, Liver, Lung, Muscle Skeletal, Ovary,Pancreas, Prostate, Skin Sun Exposed, Skin Not Sun Exposed (Suprapubic), Small Intestine Terminal Ileum, Spleen, Stomach, Substantia Nigra, Testis, Thyroid) were obtained from the GTEx consortium through dbGap (accession: phs000424)
* transcription start sites from peripheral tissue (brain, colon, esophagus, heart, kidney, liver, lung, ovary, small intestine, testis, thymus, and thyroid) were obtained through the Fantom5 consortium at ftp://ftp.ddbj.nig.ac.jp/ddbj_database/dra/fastq/DRA000/
* Human super- and typical-enhancer coordinates were downloaded from the human super-enhancer database (SEdb) at http://www.licpathway.net/sedb/

Field-specific reporting

Please select the one below that is the best fit for your research. If you are not sure, read the appropriate sections before making your selection.

[x] Life sciences [ ] Behavioural & social sciences [ ] Ecological, evolutionary & environmental sciences

For a reference copy of the document with all sections, see nature.com/documents/nr-reporting-summary-flat.pdf

Life sciences study design

All studies must disclose on these points even when the disclosure is negative.

Sample size	The sample size was determined by saturation analysis of transcription start sites (TSS). This allowed us to estimate the number of start sites we can detect at current sample size and sequencing depth.
Data exclusions	No data were excluded from the analyses
Replication	There are five biological replicates for each mTEC population
Randomization	We studied gene expression in the human thymic epithelium based on healthy samples; no group effects or treatment effects were tested and therefore no randomization conducted
Blinding	We studied gene expression in the human thymic epithelium based on healthy samples; no group effects or treatment effects were tested and therefore no blinding was necessary

Reporting for specific materials, systems and methods

We require information from authors about some types of materials, experimental systems and methods used in many studies. Here, indicate whether each material, system or method listed is relevant to your study. If you are not sure if a list item applies to your research, read the appropriate section before selecting a response.

Materials & experimental systems

n/a	Involved in the study	
[ ]	[x]	Antibodies
[x]	[ ]	Eukaryotic cell lines
[x]	[ ]	Palaeontology and archaeology
[x]	[ ]	Animals and other organisms
[ ]	[x]	Human research participants
[x]	[ ]	Clinical data
[x]	[ ]	Dual use research of concern

Methods

n/a	Involved in the study	
[x]	[ ]	ChIP-seq
[ ]	[x]	Flow cytometry
[x]	[ ]	MRI-based neuroimaging

Antibodies

Antibodies used	* biotinylated anti-epithelial cell adhesion molecule - EpCAM/sav-PE clone HEA125 (Catalog: 130-113-264), kindly provided by Gerhard Moldenhauer, DKFZ<br>* anti-cortical dendritic reticulum antigen 2 - CDR2-Alexa488, Alexa Fluor 488Protein Labeling kit; Molecular Probes, Invitrogen, Germany<br>* Human leukocyte antigen-DR - Alexa 680-conjugated mAb HLA-DR; Alexa Fluor 680 ProteinLabeling kit; Molecular Probes; clone L243, kindly provided by Gerhard Moldenhauer, DKFZ<br>*anti-CD45- PerCP - clone2D1, BD Biosciences (Catalog No: 345809)
Validation	* anti-CD45- PerCP and Alexa 680-conjugated mAb HLA-DR: Gene coexpression in human mTECs<br>Sheena Pinto, Chloé Michel, Hannah Schmidt-Glenewinkel, Nathalie Harder, Karl Rohr, Stefan Wild, Benedikt Brors, Bruno Kyewski<br>Proceedings of the National Academy of Sciences Sep 2013, 110 (37) E3497-E3505; DOI: 10.1073/pnas.1308311110<br>*anti - CDR2-Alexa488: 19. Rouse, R.V., L.M. Bolin, J.R. Bender, and B.A. Kyewski. 1988. Monoclonal antibodies reactive with subsets of mouse and human thymic epithelial cells. J. Histochem. Cytochem. 36:1511–1517.<br>*anti - EpCAM/sav-PE clone HEA125: Biotinylation kit - https://www.thermofisher.com/order/catalog/product/21425?SID=srch-srp-21425; specificity validated by epitope competition with 7 other clonese as described by the manufacturer: https://www.miltenyibiotec.com/US-en/products/cd326-epcam-antibody-anti-human-hea-125.html#gref

Human research participants

Policy information about studies involving human research participants

Population characteristics	Five human thymus samples from patients aged 6 days to 9 months, 3 male, 2 female; all patients had non-syndromic congenital cardiac malformations.
Recruitment	Samples were obtained from children undergoing corrective cardiac surgery; recruitment was unbiased
Ethics oversight	Institutional Review Board of the University of Heidelberg

Note that full information on the approval of the study protocol must also be provided in the manuscript.

Flow Cytometry

Plots

Confirm that:

- [x] The axis labels state the marker and fluorochrome used (e.g. CD4-FITC).
- [x] The axis scales are clearly visible. Include numbers along axes only for bottom left plot of group (a 'group' is an analysis of identical markers).
- [x] All plots are contour plots with outliers or pseudocolor plots.
- [x] A numerical value for number of cells or percentage (with statistics) is provided.

Methodology

Sample preparation	Thymi were digested sequentially with three rounds of collagenase/dispase for 20 min each at 37°C, followed by trypsin for 10 min each at 37°C in a water bath with magnetic stirring. The trypsin fractions were pooled and filtered through 60µm gauze. MTECs were enriched by magnetic cell sorting followed by cell staining and FACS. Magnetic cell sorting was performed using anti-CD45 Microbeads (Miltenyi Biotech, Germany).The labeled CD45+ cells were depleted using the autoMACSTM Pro Separator (Miltenyi Biotech). The enriched stromal cell fraction (CD45-)was stained with biotinylated anti-epithelial cell adhesion molecule (EpCAM/sav-PE clone HEA125, kindly provided by Gerhard Moldenhauer, DKFZ), CDR2-Alexa488 (cortical dendritic reticulum antigen 2, DKFZ, Alexa Fluor 488Protein Labeling kit; Molecular Probes, Invitrogen, Germany), Alexa 680-conjugated mAb HLA-DR (Alexa Fluor 680 ProteinLabeling kit; Molecular Probes; clone L243, kindly provided by Gerhard Moldenhauer, DKFZ), and anti-CD45- PerCP (clone21D, BD Biosciences). MTECs were sorted as CD45-, CDR2-, EpCAM+ cells and MHCII (HLA-DR) was used to separate immature mTEClo and mature mTEChi cell populations. Dead cells were excluded with propidium iodide (0.2µg/ml). Cell sorting was performed on a FACS Aria (BD Biosciences).
Instrument	FACS Aria, BD Biosciences
Software	BD FACSDiva v8.2, FlowJo - v7.6
Cell population abundance	The purity of the FAC-sorted mTEC_hi and mTEC_lo populations was 97-99%.
Gating strategy	Forward (FSC-A) and side scatter (SSC-A) to select all cells; gate on viable (negative gate on propidium iodide), CD45- cells (everything but hematopoietic); gate on EPCAM high, CDR2 (cortical TEC marker) negative cells to select for mTECs; gate for mTEClo and mTEChi populations based on their MHCII expression.

- [x] Tick this box to confirm that a figure exemplifying the gating strategy is provided in the Supplementary Information.
